# Controlling the Conformational Changes in Donor–Acceptor [4]-Dendralenes through Intramolecular Charge-Transfer Processes

**DOI:** 10.1002/chem.200900656

**Published:** 2009-09-16

**Authors:** Alexander L Kanibolotsky, John C Forgie, Greg J McEntee, M Munsif A Talpur, Peter J Skabara, Thomas DJ Westgate, Joseph JW McDouall, Michael Auinger, Simon J Coles, Michael B Hursthouse

**Affiliations:** [a] WestCHEM, Department of Pure and Applied Chemistry, University of StrathclydeGlasgow G1 1XL (UK),; [b] On leave from: Department of Chemistry, Shah Abdul Latif UniversityKhairPur (Mir’s), Sindh (Pakistan); [c] School of Chemistry, University of ManchesterManchester M13 9PL (UK); [d] On leave from: Institute for Chemical Technology of Inorganic Materials, Johannes Kepler University LinzLinz (Austria); [e] Department of Chemistry, University of SouthamptonSouthampton SO17 1BJ (UK)

**Keywords:** conformational isomerization, crystallography, dendralenes, electrochemistry, molecular modeling

## Abstract

The synthesis of two [4]-dendralene compounds incorporating thiophene-(*p*-nitrophenyl) donor–acceptor units is presented. The dendralenes adopt two different conformers in solution and solid state and the transformation between the structures can be controlled by light and heat. The electron-donating components of the dendralenes are represented by bromothienyl (in **13**) and ethylenedioxythiophene(EDOT)-thienyl (in **15**) end-groups. The most facile transformation involves the isomerisation of donor–acceptor conjugated systems (**a** conformers) into structures in which only the thiophenes are conjugated (**b** conformers), and this process is driven by ambient light. The structures of the two conformers of compound **13** are confirmed by single-crystal X-ray diffraction studies and the structural changes in both compounds have been monitored by ^1^H NMR spectroscopy and absorption studies. The transformations were found to be first-order processes with rate constants of *k* = 0.0027 s^−1^ and *k* = 0.00022 s^−1^ for **13** and **15**, respectively. Density functional theory calculations at the B3LYP/6-31G∗ level give credence to the proposed mechanism for the **a**→**b** conversion, which involves photoinduced intramolecular charge transfer (ICT) as the key step. The EDOT derivative (**15**) can be polymerised by electrochemical oxidation and a combination of cyclic voltammetry and UV/Vis spectroelectrochemical experiments indicate that the **a** conformer can be trapped and stabilised in the solid state.

## Introduction

Cross-conjugated molecules have been defined as containing three or more unsaturated groups, two of which are conjugated to the third but not directly to each other.^1^ For example, in benzophenone the two phenyl groups share conjugation to the carbonyl group, but are not conjugated to each other. Similarly, in 2-methylenemalonic acid the carbonyl groups are conjugated to the central C=C group, but not to each other.^1^ This definition is not entirely unambiguous, as it could be said to hold for linear *π*-conjugated systems.^2^ The definition can be expanded, however, to structures incorporating two or more unsaturated groups that are all conjugated to a central unsaturated core group, but not directly conjugated to each other.

**Scheme 1:**
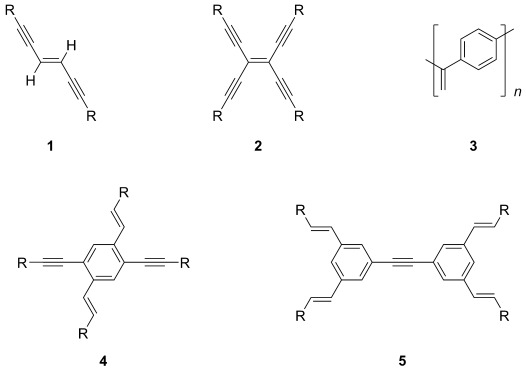
Some conjugated and cross-conjugated motifs that have been investigated in molecular electronics: dialkynylethene 1, tetraalkynylethene 2, oligo(*p*-phenylenevinylidene) 3, tetra-substituted benzene cruciform 4 and tetra-substituted tolane cruciform 5. R=alkyl or aryl group.

Interest in cross-conjugated structures as candidates for electronic components has developed because they frequently provide the opportunity to investigate the fundamental relationships between *π*-electron delocalisation, conformation and optoelectronic properties. Scheme 1 shows some conjugated motifs that have been well studied in this area: dialkynylethene (**1**),^3^–^10^ tetraalkynylethene (**2**),^11^–^16^ oligo(*p*-phenylenevinylidene) (**3**)^2^ and cruciforms constructed around a tetra-substituted benzene (**4**)^17^–^34^ or tolane core (**5**).^35^

Several properties of these structures that are beneficial or desirable in optical or electronic systems have been identified. Cross-conjugation allows for an extension of the conjugated system without the corresponding bathochromic shift of absorption wavelength associated with linear conjugation. This leads to interesting candidates for optoelectronic applications that rely on transparency in the visible and near infrared regions, such as nonlinear optics.^2^,^9^,^27^ The opposite can be achieved by appending multiple chromophores at the cross-conjugated arms, thus engineering a broader absorption profile desirable in photovoltaic devices.^35^ Multiple conduction pathways in different dimensions are accessible by using cross-conjugated compounds.^20^–^22^,^29^,^30^ Electron-donor and -acceptor moieties are frequently incorporated into cross-conjugated structures to improve the dimensionality of electronic communication.^4^,^5^,^10^,^22^,^27^,^36^–^40^ Judicial placement of the donor and acceptor groups allows spatial separation of the HOMO and LUMO, as revealed by quantum-mechanical calculations. In turn, this arrangement allows the optical band gap to be tailored, and either of the frontier orbitals to be addressed independently, for example, with host–guest interactions.^17^,^19^,^22^,^25^,^26^,^29^–^31^

**Scheme 2:**
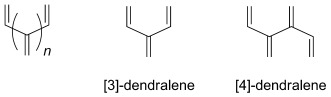
Generic and sample structures of the dendralene family of compounds.

Dendralenes^41^–^43^ are a family of cross-conjugated compounds that contain the motifs shown in Scheme 2. Dendralenes are named according to the number of C=C groups in this unit, so [3]-dendralene has *n*=1, [4]-dendralene has *n*=2, and so on. Many of the properties described above are common to all the cross-conjugated systems but research into the synthetic versatility of dendralenes in cycloaddition procedures^44^–^47^ has taken prominence recently over interest in their electronic and structural properties. However, the development of analogues of tetrathiafulvalene with conjugated spacer groups separating two or more 1,3-dichalcole groups has yielded a few redox-active dendralene structures, including for example **6** and **7**.^48^–^53^
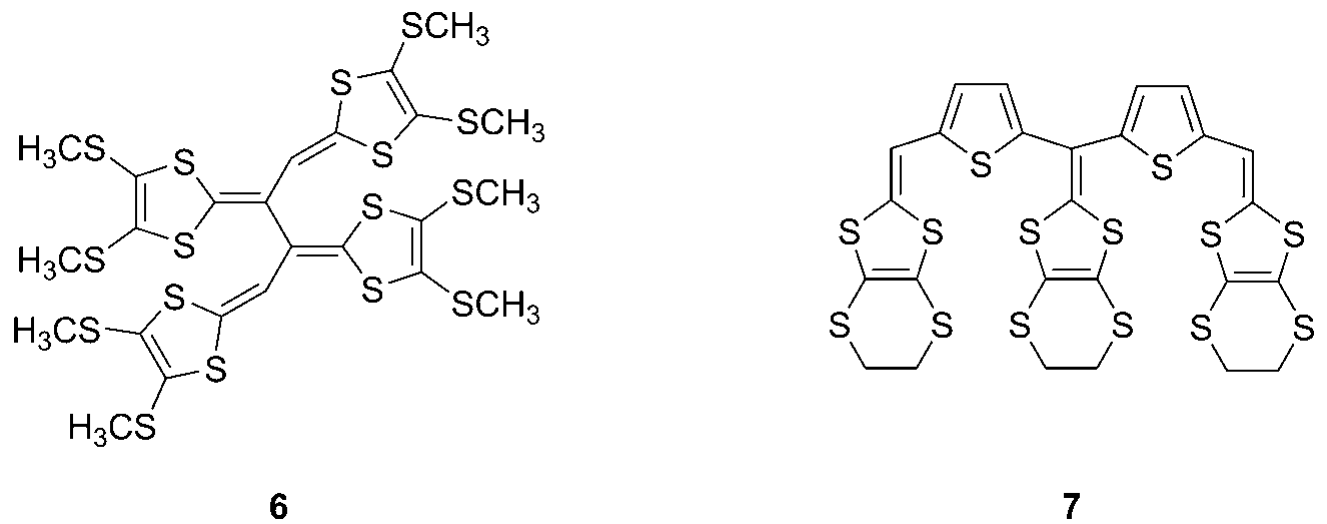


The C-C sigma bonds impart conformational flexibility on the dendralenes where steric factors allow for it.^54^,^55^ Some studies on chalcogen-containing dendralenes have highlighted the conformational variability in structures such as **8**
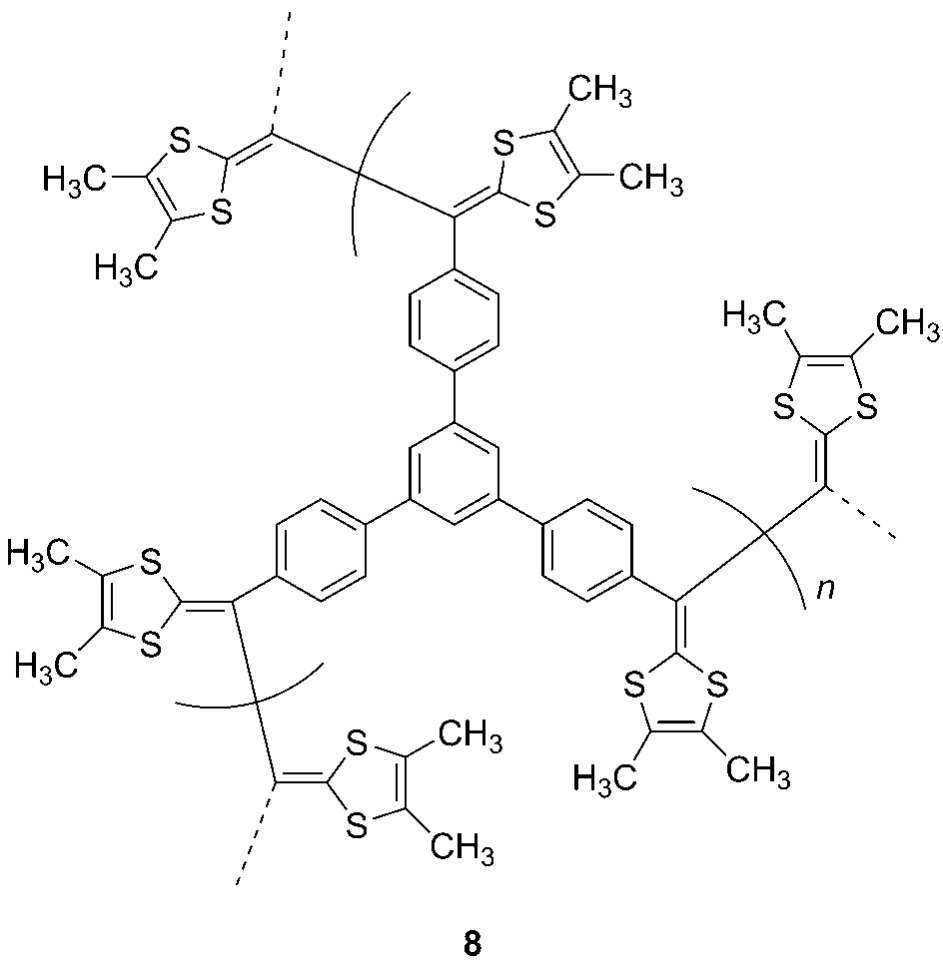
while others have demonstrated electrochemical control over conformation.^[^56–58^]^ Controlled molecular motion, in conjunction with controlled intramolecular electronic pathways, are fascinating and potentially useful features in the design of optical switching components and molecular machines.^59^–^62^ In this paper, we report a novel synthetic route to a donor–acceptor dendralene that undergoes a reversible conformational change driven by light and heat. This is an unusual process, given that the two structures appear to be “conformationally locked”. The extension of this dendralene with ethylenedioxythiophene (EDOT) units provides an electropolymerisable derivative. Whilst photoinduced *trans*–*cis* isomerism has been reported for some donor–acceptor 1,2-diethynylethenes and tetraethynylethenes,^63^ our dendralenes could be adapted in macromolecular structures to give short or long conjugation lengths. The electronic properties of such macromolecules would be vastly different between isomeric structures and the construction of these materials could be manipulated by photoexcitation, providing a valuable and unique method for structural and electronic control. Herein, we report the spectroscopic and electrochemical properties of donor–acceptor dendralenes and relate these results to the conformational changes observed under external stimuli.

## Results and Discussion

**Scheme 3:**
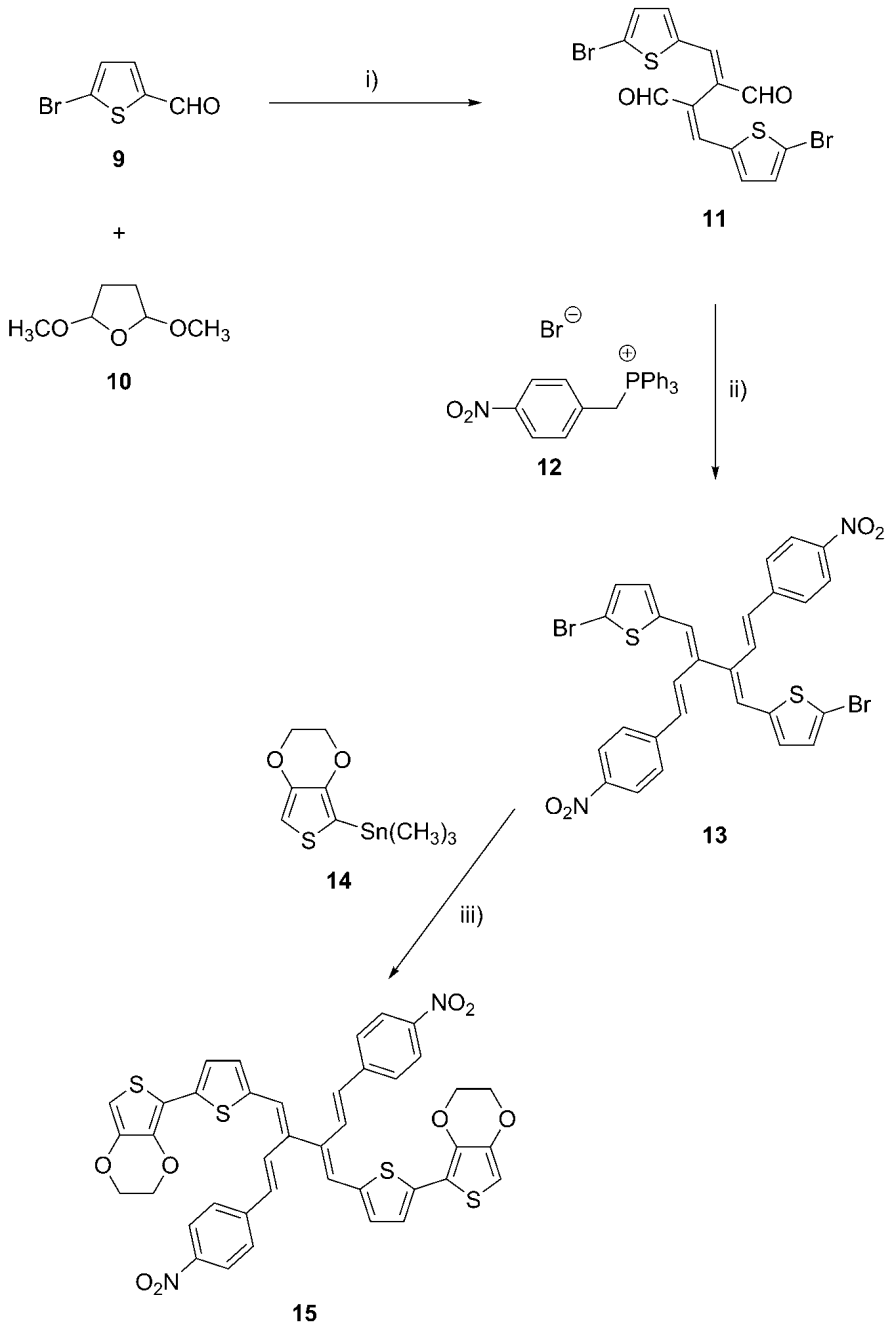
Synthesis of dendralenes 13 and 15: i) potassium acetate, acetic acid, water, heating to reflux, 16 h; ii) potassium *tert*-butoxide, THF, heating to reflux, 2 days; iii) [Pd(PPh_3_)_4_], DMF, microwave, 160°C, 1 h.

**Synthesis**: The synthetic route towards [4]-dendralenes **13** and **15** is summarised in Scheme 3. Compound **11** was prepared in 22 % yield from 5-bromothiophene-2-carboxaldehyde (**9**) and 2,5-dimethoxytetrahydrofuran (**10**) by heating at reflux in the presence of potassium acetate, acetic acid and water.^64^,^65^ Dendralene **13** was synthesised by a two-fold Wittig reaction from **11** with *p*-nitrobenzyl triphenylphosphonium bromide (**12**) and potassium *tert*-butoxide under reflux THF (33 % yield). Coupling of dendralene **13** with 2-trimethylstannyl-3,4-ethylenedioxythiophene (**14**) under Stille conditions was achieved by using DMF as the solvent and microwave assisted heating. Dendralene **15** was obtained in 73 % yield.

**Scheme 4:**
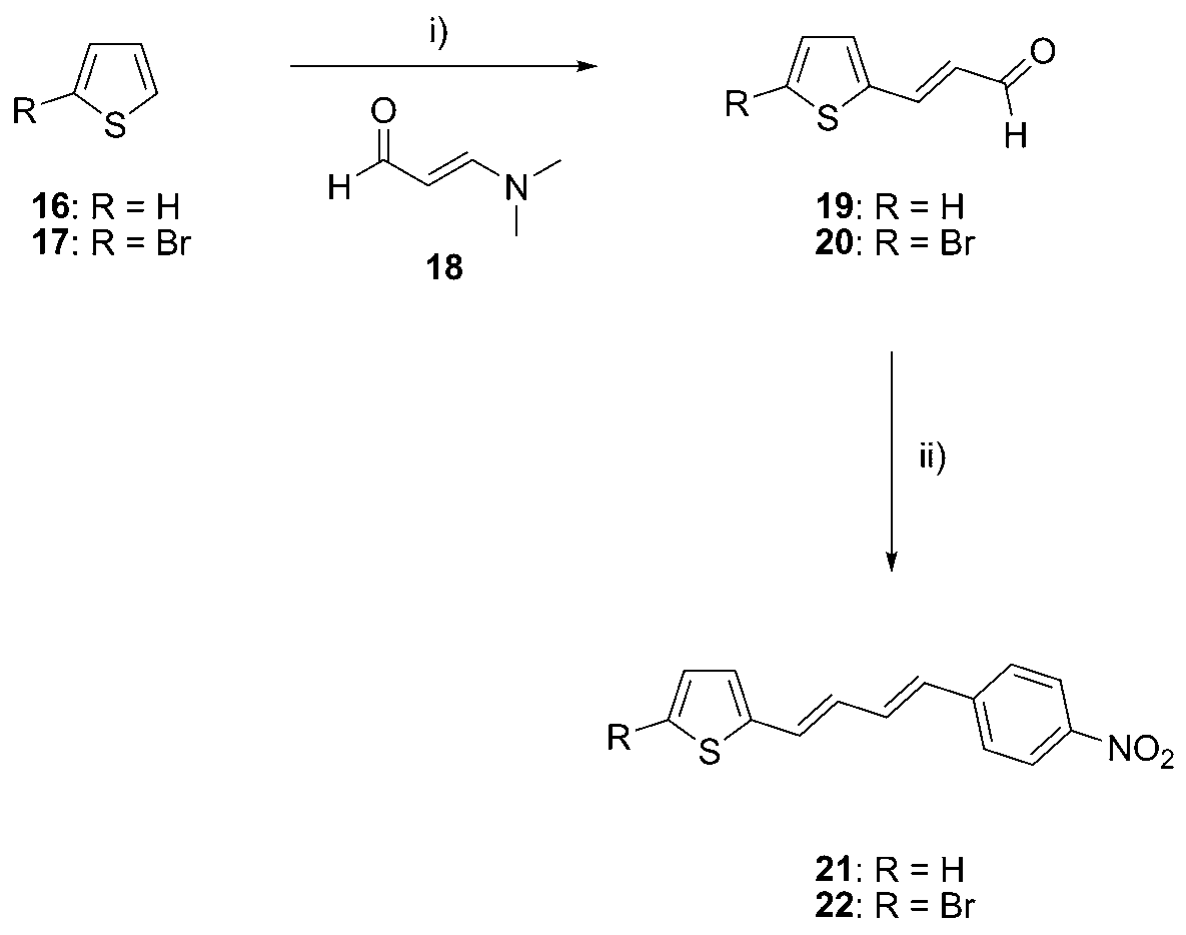
Synthesis of compounds 21 and 22: i) lithium diisopropylamide (LDA), THF, −78 °C, 45–60 min, then (*E*)-3-(dimethylamino)acrolein (18); ii) potassium *tert*-butoxide, THF, 30 min, then (*p*-nitrobenzyl)triphenylphosphonium bromide (12), 16 h.

Compounds **21** and **22** (Scheme 4) were prepared as analogous donor–*π* acceptor units for comparison with the donor–acceptor characteristics of dendralene **13**. Reaction of 2-lithiothiophene or 2-bromo-5-lithiothiophene (generated in situ from **16** or **17**) with 2-(*N*,*N*-dimethylamino)acrolein (**18**) afforded the aldehydes **19** (18 % yield) or **20** (24 % yield), which were isolated in *trans* geometry after distillation.^66^ Reaction of **19** or **20** with (*p*-nitrobenzyl)triphenylphosphonium bromide (**12**) and potassium *tert*-butoxide yielded **21** (19 % yield) or **22** (56 % yield). In both cases, a mixture containing *cis* and *trans* geometries at the conjugated double bonds was generated. The all-*trans* isomer was isolated by crystallisation from dichloromethane and petroleum ether; the isomers were verified by NMR and, in the case of **22**, X-ray crystallography.

**Figure 1:**
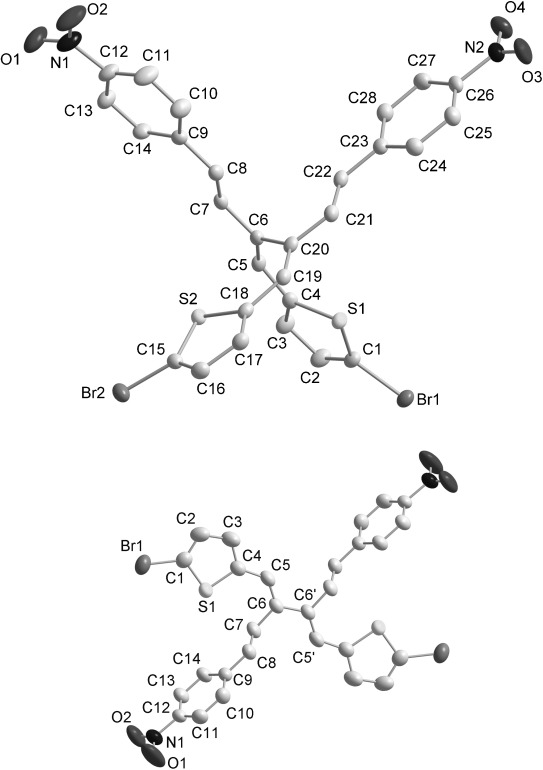
X-ray crystal structure of conformers 13 a (top) and 13 b (bottom).

**X-ray crystallography**: Crystalline samples of **13** were prepared from the mixture of conformers by: i) Dissolution in a minimum amount of warm, degassed dichloromethane under nitrogen, followed by the addition of degassed hexane to form a cloudy, yellow solution. The crystals were then allowed to grow at room temperature in the dark. ii) Slow diffusion of hexane into a solution of **13** in dichloromethane at room temperature and exposed to ambient light. The two methods gave different X-ray crystal structures, which are shown in Figure 1. Crystals grown by method i) were found to have the structure **13 a** (Figure 1, top), which consists of two orthogonal and identical donor–*π*-acceptor units forming a cruciform shape. Each bromothiophene is conjugated to a nitrobenzene unit through an all-*trans* butadiene bridge (C5-C6-C7-C8 and C19-C20-C21-C22) and the torsion angle between the two donor–*π*-acceptor units (C7-C6-C20-C21) is 83.03(3)°. The single bond linking the two orthogonal units (C6-C20) is weakly conjugated with a C-C bond length of 1.497(3) Å. Crystals grown by method ii) gave the conformer represented by **13 b** (Figure 1, bottom). Here, the bromothiophene units are co-planar and conjugated through an all-*trans* butadiene bridge (C5-C6-C6′-C5′) and the nitrobenzene units are twisted out of the plane of the bromothiophenes with a torsion angle of 99.94(4)° (C5-C6-C7-C8). The carbon-carbon bond linking the styryl units to the butadiene fragment (C6-C7) has a length of 1.481(4) Å, again showing weak conjugation to the planar butadiene chain. The C=C bonds between the carbons α and β to the bromothiophene (C5/C6 and C19/C20 in **13 a**) that possessed *cis* geometry in **13 a** have changed to a *trans* geometry in **13 b** (C5/C6 in **13 b**). The C-C bond length alternation gives a measure of the extent of conjugation in each structure above. In a highly conjugated structure, single and double bonds will tend to become equivalent due to extensive *π*-delocalisation. Within the conjugated butadiene chain of **13 a**, there is a notable difference of 0.108 Å between the average C-C bond length and the average C=C bond length. In **13 b**, the bond length alternation is 0.109 Å. This suggests that in both conformers, the C=C double bonds are quite localised. The conjugated segments in both conformers provide good coplanarity between terminal aryl units with a deviation from planarity of 0.008(3) Å for **13 a** (thiophene–benzene) and 0.059(3) Å for **13 b** (thiophene–thiophene).

**Figure 2:**
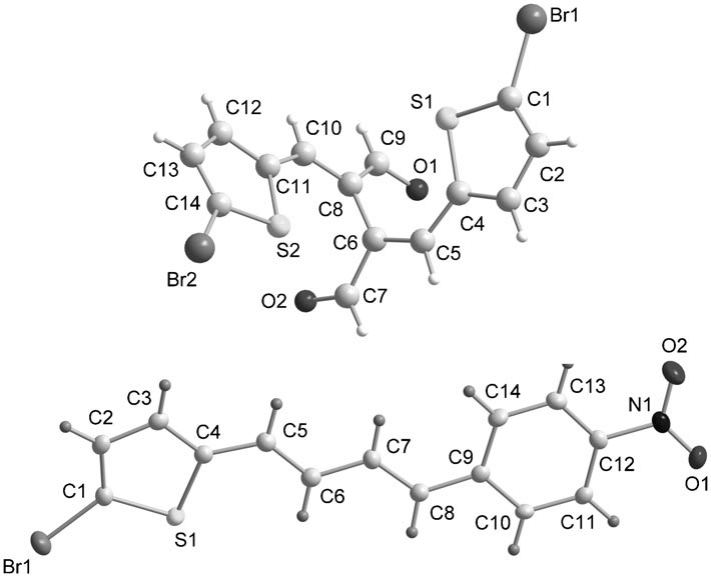
X-ray crystal structures of compounds 11 (top) and 22 (bottom).

Slow diffusion of hexane into a solution of **11** in chloroform gave suitable samples for X-ray crystallography. The crystal structure of **11** is shown in Figure 2 (top). The preferred conformation in this molecule presents two orthogonal donor–acceptor half-units, which are twisted at approximately 99.06(4)° (C5-C6-C8-C10) and joined by a C-C single bond (C6-C8, 1.492(4) Å). This arrangement is the same as that reported for other 1,4-diaryl-2,3-diformylbutadienes where the aryl groups are alkyl- and alkoxy-substituted phenylene rings.^64^ Polymorphs were not obtained by varying the conditions of crystal growth (air and light). As a useful comparison to conformer **13 a**, the crystal structure of compound **22** was also obtained (see Figure 2, bottom). The bond length alternation in **22** can be compared with that of the corresponding dendralene. The difference between the average C-C and C=C bond lengths in **22** is 0.099 Å, a value very close to that in **13 a**. This suggests there is little effect on the conjugation between the bromothiophene and nitrobenzene units caused by the presence of a second donor–acceptor arm in **13 a**.

The different methods of crystallisation for compound **13** provide two distinct conformers. In **13 a**, the molecule adopts a common dendralene motif, in which two conjugated half-units give a cruciform structure with a single non-conjugated node. Conformer **13 b**, on the other hand, provides a different scenario, in which the conjugated fragment is a 1,4-di(thiophen-2-yl)buta-1,3-diene, substituted at the 2,3-positions by twisted nitrostyryl units and resulting in two non-conjugated nodes within the structure. From the different crystallisation methods applied for the generation of each unique structure and the reproducibility of these experiments, we suspected that external physical factors could be used to enforce geometrical transformations between the two conformers. The following sections evaluate the conversion of **13 a** into **13 b** under ambient light and, on the basis of our results, we propose a mechanism that would explain this fascinating transformation.

**Solution-state conversion between conformers 13 a and 13 b**: Given that two molecular conformations are accessible for **13** in the crystal phase, it is logical to assume that dendralenes of this type are conformationally flexible in solution. Indeed, it was possible to monitor the transformation between the **a** and **b** conformers by using solution-state spectroscopy.

**Figure 3:**
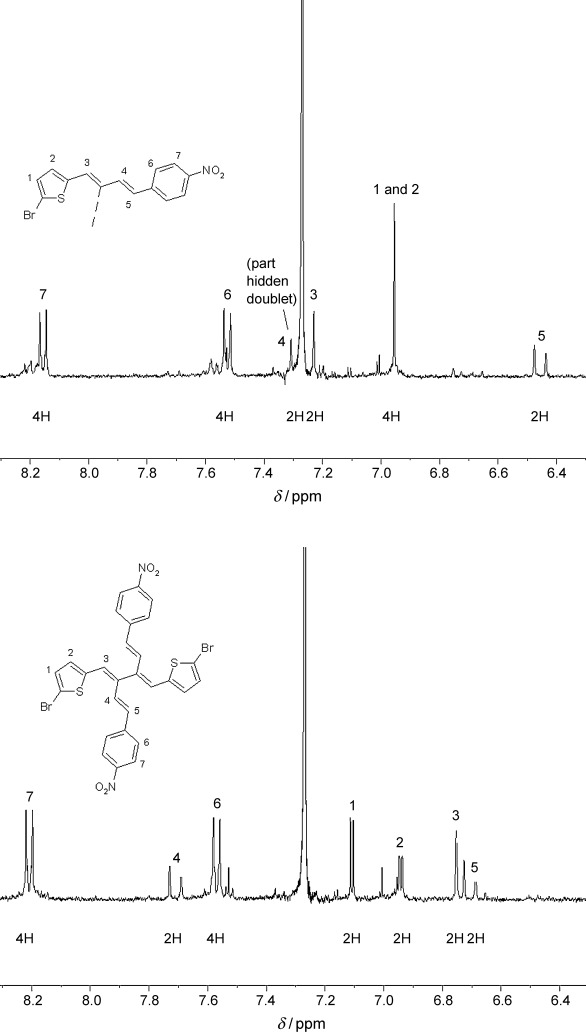
Top) ^1^H NMR spectrum of 13 a in the region *δ*=6.2–8.5 ppm. Bottom) ^1^H NMR spectrum of the same solution after 24 h exposure to ambient light.

^**1**^**H NMR spectroscopy**: The two forms of **13** can be identified by proton NMR in CDCl_3_ and in [D_6_]DMSO. Crystals from the same batch as those that had been shown by X-ray crystallography to have the conformation **13 a** were dissolved in CDCl_3_ in the absence of light. The ^1^H NMR spectrum was immediately collected from this solution. The solution was only exposed to light for a few seconds as the NMR tube was transferred to the spectrometer magnet. Figure 3 a shows the spectrum obtained in this manner, which confirms that the structure seen in **13 a** crystals persists in solution. The solution was then exposed to ambient light for 24 h and the proton NMR spectrum was recorded again. This spectrum is shown in Figure 3 b and can be assigned to the molecular conformation of **13 b**. The signals for **13 b** can in fact be seen emerging in the spectrum of **13 a** as a result of the short exposure to light that occurred between sample preparation and data acquisition. The nitrobenzene proton signals of **13 a** (arising from positions 6 and 7 in Figure 3 a, *δ*=8.16 and 7.53 ppm) become shifted to slightly lower field (*δ*=8.21 and 7.57 ppm) in **13 b**. The coupling constant between these two protons (*J*=8.8 Hz) remains unchanged. The protons of the styryl C=C positions are similarly shifted; the signal from proton 4 shifts from *δ*=7.29 ppm to *δ*=7.71 ppm, and the signal from proton 5 shifts from *δ*=6.46 ppm to *δ*=6.71 ppm. The coupling constant arising from the *trans*-vinyl relationship between 5 and 4 (seen in the 5 peak, obscured by solvent in the 4 peak) remains unchanged at *δ*=15.7 Hz. This downfield shift of the signals can be attributed to the lack of conjugation from the electron-donating bromothiophene group allowing for the electron-withdrawing effect of the nitrobenzene group to dominate and deshield the protons. In contrast, the proton in position 3 loses the deshielding effect of a conjugated nitrobenzene group and its singlet peak shifts to higher field (from *δ*=7.23 ppm to *δ*=6.75 ppm) as its position becomes conjugated to a second electron-donating bromothiophene group. The thiophene signals are coalesced in **13 a**, possibly as a result of the donor–acceptor sequence, but resolve into separate peaks in **13 b**.

During this process of conversion, transient intermediate signals can be seen in the NMR spectrum if it is recorded within 24 h. Virtually, all of these signals disappear once conversion is complete and their relative intensity is dependent on the progress of the transformation. A few minor impurities remain in the spectrum of **13 b** and are likely to represent more persistent intermediates of the transformation process. It should also be noted that if no light is allowed to the solution at any time during the experiment then the NMR spectra remain unchanged.

The same effect can be seen if the solution is prepared by using [D_6_]DMSO as the NMR solvent. Analogous to the CDCl_3_ experiment, crystals from a batch of conformer **13 a** were dissolved in [D_6_]DMSO and the proton NMR was recorded taking the same precautions to exclude light from the solution as much as possible (see Figure S1 a in the Supporting Information). The solution was then exposed to ambient light for 24 h and the spectrum was recorded again (see Figure S1 b in the Supporting Information), allowing identification of conformer **13 b**. This solution was then transferred to a microwave reaction vial and heated to 120 °C for 40 h. The cooled solution was then transferred to an NMR tube and the proton NMR spectrum was recorded taking care to minimise exposure to ambient light (see Figure S2 in the Supporting Information). This spectrum indicated that the original **13 a** form was now the predominant component of the mixture, proving that the conformational conversion is reversible. Conversion was not 100 % complete, however, and some intermediate signals were still present. A repeat of the process on the same solution with heating for an additional 6 h returned a poor quality spectrum, possibly as a result of decomposition during heating, and from absorption of water by the solvent.

^1^H NMR and UV/Vis analysis of **15** revealed that the two components observed by TLC on silica are also two conformational forms of the same type as **13**. Because the transformation in this case was much slower, it was possible to isolate the components of **15** by column chromatography and identify the pure **a** form, possessing donor–acceptor conjugation, by ^1^H NMR spectroscopy. It was not possible to identify and fully characterise the **b** conformer as the process of conversion was incomplete.

**UV/Vis absorption studies**: The UV/Vis absorption spectra of compounds **1321** and **22** were recorded. Compounds **21** and **22** have almost identical spectra; they have a maximum absorbance at *λ*=395 nm and *λ*=397 nm, respectively, and a shoulder at approximately 320 nm (see Figure S3 in the Supporting Information). The major absorbance can be assigned to a “push-pull” intramolecular charge transfer (ICT) sequence from electron-donor (thiophene) to -acceptor (nitrobenzene) units. Since the absorption maxima of **21** and **22** are so similar, it can be surmised that there is no significant affect caused by the presence of bromine on the energy of this transition. The molar extinction coefficient, however, is larger (*ε*=43 000 m^−1^ cm^−1^) for the bromothiophene derivative **22**, compared to **21** (*ε*=33 000 M^−1^ cm^−1^).

**Figure 4:**
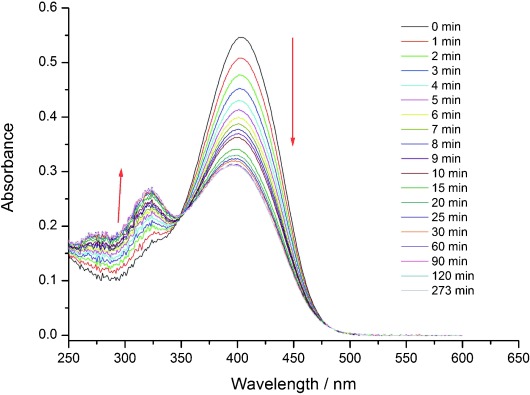
Evolution of UV/Vis absorption spectra of a solution of 13 a in CH_2_Cl_2_ over time when exposed to ambient light.

The conversion of **13 a** to **13 b** can be observed by UV/Vis spectroscopy. Crystals of structure **13 a** (selected as above) were dissolved in dry CH_2_Cl_2_ in the absence of light, then immediately transferred to the UV/Vis spectrometer. The spectrum of the fresh solution is very similar to the analogous compounds **21** and **22**. The maximum absorbance occurs at approximately *λ*=403 nm and is also ascribed to ICT. If natural light is allowed to irradiate the sample, the intensity of the longest wavelength band decreases gradually and is slightly blue-shifted to *λ*=397 nm (Figure 4), whilst a peak at *λ*=320 nm develops. This peak is noisy due to its low intensity, but an isosbestic point can clearly be seen in Figure 4. If no light is allowed to the solution, the spectrum is repeatable at any point during the process. The same effect can be seen when chloroform is used instead of dichloromethane as the solvent.

**Figure 5:**
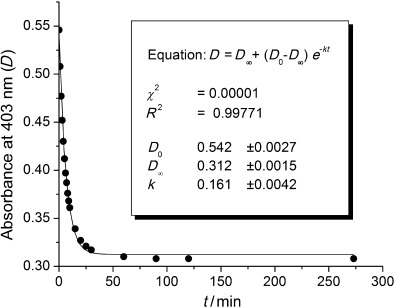
Kinetics plot of the rate of change in the absorbance of a solution of 13 in CH_2_Cl_2_ at *λ*=403 nm with time, upon exposure to ambient light.

The rate of change of the absorbance at *λ*=403 nm is shown in Figure 5. The data are a good fit to the first-order rate law, which is solved for a first order rate constant *k*=0.16 min^−1^, or 0.0027 s^−1^. The presence of the isosbestic point reinforces the stable first-order kinetic behaviour. The levels of noise in the peak at *λ*=320 nm render any examination of the rate of change in intensity of this band inaccurate.

A sample of **13 b** was obtained by removing the CDCl_3_ solvent in vacuo from a solution that had been shown by NMR to contain predominantly that conformer. The resultant solid was dissolved in dichloromethane and left in ambient light for one hour to ensure that any **13 a** conformer could convert to **13 b**. The UV/Vis spectrum of this solution was then collected (see Figure S4 in the Supporting Information). This spectrum is practically identical to the one obtained after sufficient time had elapsed in the conversion experiment detailed above. The *λ*_max_ is seen at 395 nm, with two higher energy peaks at *λ*=320 nm and *λ*=270 nm.

**Figure 6:**
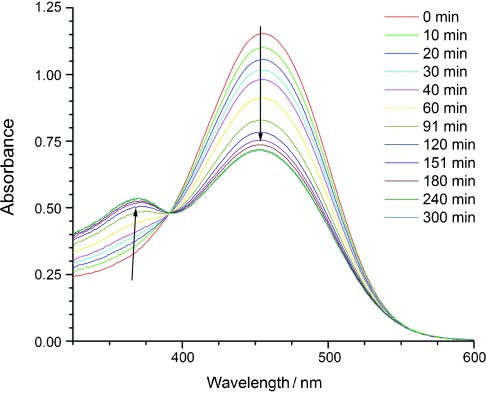
Evolution of UV/Vis absorption spectra of a solution of 15 in CH_2_Cl_2_ over time when exposed to ambient light.

A similar effect can be observed in dendralene **15**. A solution of a sample of **15** that had been identified by ^1^H NMR as having the donor–acceptor coplanar conformation was dissolved in dry CH_2_Cl_2_ in the absence of light. Absorption spectra were recorded over time with exposure to ambient light in the same fashion as for **13** detailed above. The spectra are shown in Figure 6. The fresh solution before exposure to light gives a maximum absorption at *λ*=455 nm. Note the bathochromic shift (52 nm) relative to **13 a** resulting from the incorporation of the EDOT group into the dendralene *π* system. Exposure to light causes the intensity of this band to decrease, and a higher energy absorption band at *λ*=368 nm emerges and becomes more intense. After 300 min exposure to ambient light the spectrum ceases to change and exhibits an absorption maximum at *λ*=453 nm.

**Figure 7:**
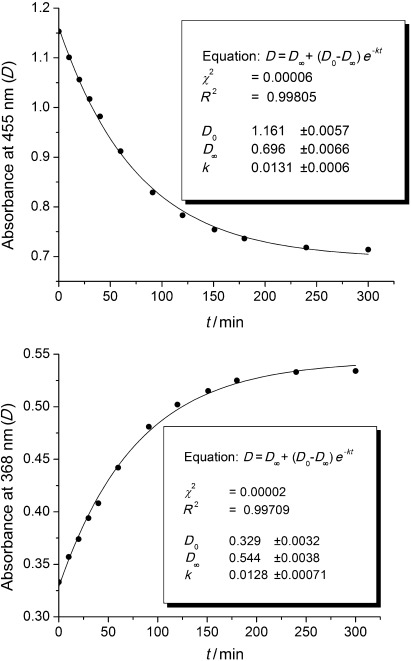
Top) Kinetics plot of the rate of change in the absorbance of a solution of 15 in CH_2_Cl_2_ at *λ*=455 nm with time, upon exposure to ambient light. Bottom) Kinetics plot of the rate of change in the absorbance of 15 at *λ*=368 nm with time, upon exposure to ambient light.

The kinetics of this process were compared with **13**. Figure 7 shows the rate of change of the absorbance at *λ*=455 nm (top), and the rate of change of the absorbance at *λ*=368 nm (bottom). The data are a good fit to the first-order rate law. The value of the first order rate constant for the diminishing band at *λ*=455 nm (*k*=0.013 min^−1^, 2.2×10^−4^ s^−1^) is equal to the rate constant of the emerging peak centred at *λ*=368 nm, enforcing the argument for first-order kinetics and proving that the contribution of by-processes to the consumption of **15** is negligible. The value of *k* for dendralene **15** is an order of magnitude lower than that for the same process in dendralene **13** (*k*=0.0027 s^−1^).

**Figure 8:**
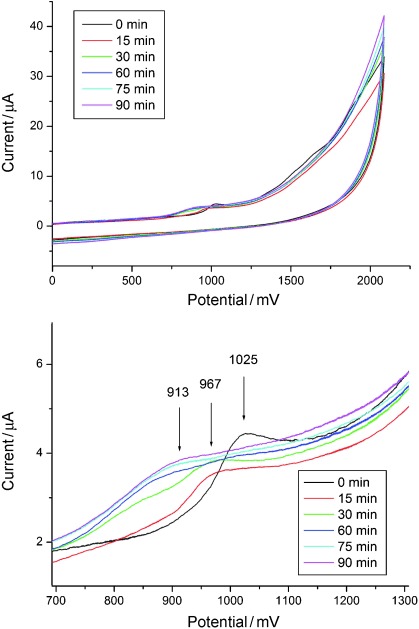
Change in cyclic voltammograms of 13 a over time whilst exposed to ambient light. Top) Full scan. Bottom) Expansion of thiophene oxidation peak. Glassy carbon working electrode, Pt wire counter and silver wire pseudo reference electrodes in CH_2_Cl_2_ (substrate concentration about 10^−4^ m) containing *n*Bu_4_NPF_6_ as supporting electrolyte (0.1 m), scan rate=100 mV s^−1^. Potentials are referenced to ferrocene as internal standard.

**Electrochemistry**: Cyclic voltammetry (CV) experiments were conducted to probe the electrochemical properties of the dendralenes and, in the case of **15**, polymerisation under oxidative conditions and the properties of the resulting material. A solution of **13 a** (10^−4^ m) and supporting electrolyte (tetra-*n*-butylammonium hexafluorophosphate, 0.1 m) in dry CH_2_Cl_2_ was prepared in the absence of light, and the cyclic voltammogram was recorded by using a glassy carbon working electrode, Pt wire counter electrode and silver wire pseudo reference electrode. The solution was then exposed to ambient light and the cyclic voltammogram was repeatedly recorded after several 15 min intervals. The cyclic voltammograms are shown in Figure 8.

The oxidation of the bromothiophenes in **13 a** is shown by a peak at +1025 mV, which shifts gradually to lower potentials as the solution is exposed to light and finally stabilises at +913 mV. As **13 a** converts to **13 b** the two bromothiophenes change from each being independently conjugated to a strongly electron-withdrawing group (nitrobenzyl) to being conjugated to each other. The absence of a conjugated electron-withdrawing group renders the removal of a bromothiophene electron less demanding, and this is consistent with the observed shift of the oxidation potential to lower energy.

Cyclic voltammetry of dendralene **15** in dichloromethane was performed in the absence of light, in the same fashion as **13** (see Figure S5 in the Supporting Information). The main feature is the oxidation of the EDOT group to form a radical cation at +465 mV. A second oxidation process is observed at higher potential (+1499 mV), but considering that the two EDOT–thiophene units are not conjugated in this dendralene structure, it is assumed that the wave at +465 mV represents the simultaneous oxidation of both EDOT–thiophene fragments. The cyclic voltammogram representing the reduction of **15** is shown in Figure S6. The first, reversible reduction wave occurs at a half-wave potential at −1403 mV and the second, irreversible reduction occurs at −1822 mV. The final reduction, resulting in the irreversible formation of a radical trianion, occurs at −2273 mV.

**Figure 9:**
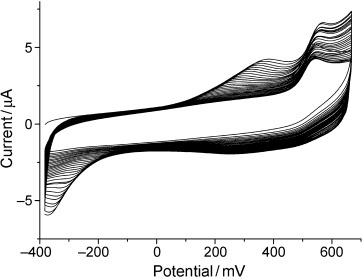
Electropolymerisation of 15 on glassy carbon working electrode, by using Pt gauze counter and silver wire pseudo reference electrodes, in CH_2_Cl_2_ (substrate concentration about 10^−4^ m) containing *n*Bu_4_NPF_6_ as supporting electrolyte (0.1 m), scan rate=100 mV s^−1^. Potentials are referenced to ferrocene as internal standard.

**Figure 10:**
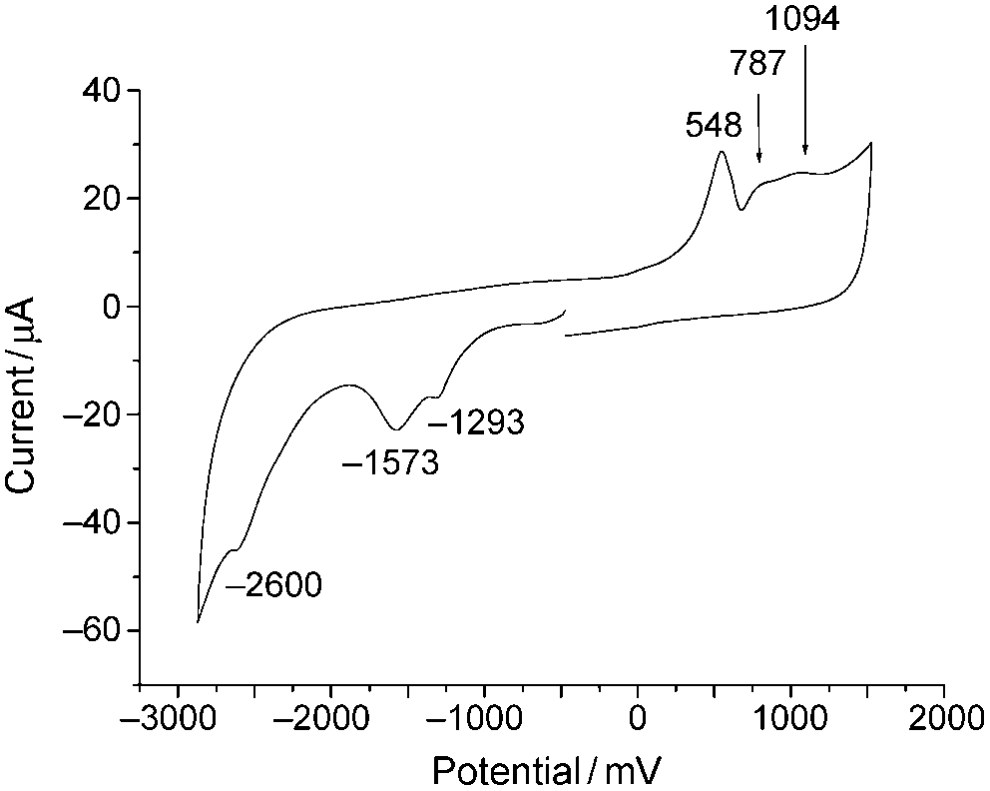
Reduction and oxidation of poly(15) deposited on glassy carbon working electrode, by using Pt counter and silver wire pseudo reference electrodes, in monomer-free acetonitrile containing *n*Bu_4_NPF_6_ as supporting electrolyte (0.1 m), scan rate=100 mV s^−1^. Potentials are referenced to ferrocene as internal standard.

Repeated cycling over the oxidation wave corresponding to the EDOT–thiophene group (between −380 mV and +670 mV, 30 cycles), caused the deposition of a film of poly(**15**) on the glassy carbon working electrode. The electrochemical growth of the film can be seen in Figure 9. The development of a new peak in the range +200 to +400 mV indicates the electroactive character of the thiophene-EDOT-EDOT-thiophene segment within the polymer backbone. The cyclic voltammogram showing oxidation of the polymer film adhered to the working electrode in monomer-free electrolyte solution is shown in Figure S7 in the Supporting Information. The main feature is the oxidation of the thiophene-EDOT-EDOT-thiophene segment in the polymer backbone, at +265 mV. A cyclic voltammogram of a film of poly(**15**) showing oxidation and reduction processes is shown in Figure 10. This scan was performed in the negative direction first so as to reduce interference from oxygen. However, despite bubbling argon into the solution for 20 min, oxygen reduction was still observed as a peak at −1293 mV. This was confirmed as an oxygen peak by running sequential differential pulse voltammetry measurements on a fresh polymer film. In this more sensitive method, the peak at −1293 mV became more intense in each scan indicating that it is caused by oxygen. Performing the reduction cycle first also had the effect of raising the potential of the first oxidation to +548 mV, indicating that the reductive conditions degrade the polymer. The multiple oxidation peaks at +548, +787 and 1094 mV are due to the formation of polarons and bipolarons in the polymer chain.

The electrochemical band gap of poly(**15**) was deduced from the full-scan cyclic voltammogram by the difference between the onset of the first reduction and oxidation waves; HOMO/LUMO energy values were measured relative to ferrocene (see Figure S8 in the Supporting Information). The electrochemical band gap of poly(**15**) was thus calculated as 1.72 eV.

Cyclic voltammograms of a film of poly(**15**), recorded over a range of scan rates, are shown in Figure S9 in the Supporting Information, together with the corresponding plot of scan rate versus maximum current of the oxidation process. In Figure S9 in the Supporting Information the peak maximum shifts to higher potentials with higher scan rate, which is a result of the oxidation being irreversible; this figure also shows a linear relationship of scan rate versus peak current, concluding that the oxidation process of the polymer is not diffusion limited.^67^

A film of poly(**15**) was grown on ITO-coated glass and the UV/Vis spectrum recorded. Figure S10 in the Supporting Information shows the spectrum of the film immediately after growth, in the doped state. There are absorption maxima at *λ*=337, 424, 569 and 759 nm. The minor peak at *λ*=759 nm indicates a low level of doping and the transition is due to the charge transfer of polarons and bipolarons from one chain to another. Figure S11 in the Supporting Information shows the absorption spectrum recorded after de-doping the film by cycling between −0.1 V and +0.1 V at 100 mV s^−1^ for one hour. The absorption maxima at *λ*=337, 425 and 569 nm remain but the peak at *λ*=759 nm is absent as a result of complete de-doping. The onset of the longest wavelength peak in Figure S11, which was found to occur at *λ*=690 nm, was used to estimate the optical band gap of the polymer at 1.80 eV. This is in good agreement with the electrochemically calculated band gap (1.72 eV).

**Figure 11:**
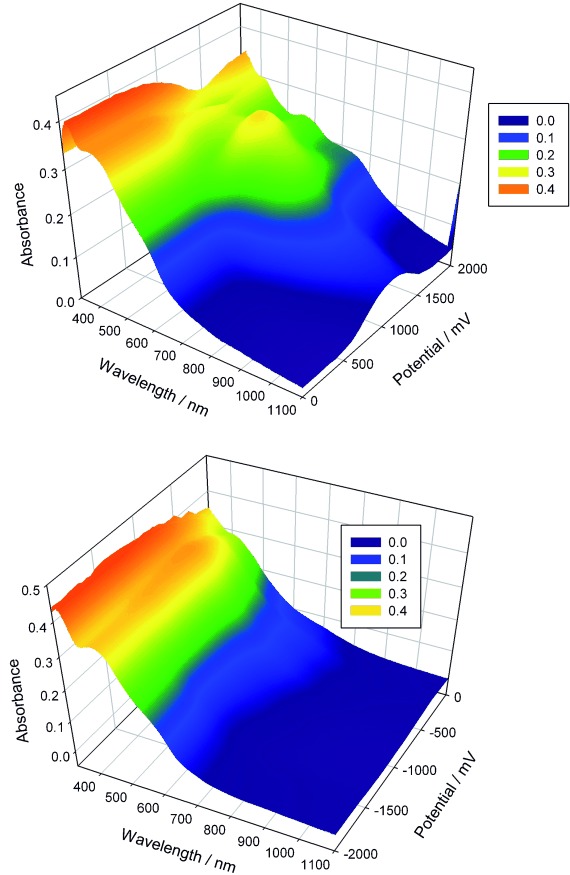
Top) Oxidative spectroelectrochemistry of poly(15) on ITO-coated glass working electrode, by using Pt wire counter and silver wire pseudo reference electrodes, in monomer-free acetonitrile containing *n*Bu_4_NPF_6_ as supporting electrolyte (0.1 m). Bottom) Reductive spectroelectrochemistry of poly(15).

UV/Vis spectroelectrochemistry was performed on a film of de-doped poly(**15**) on indium tin oxide (ITO)-coated glass. The absorption spectra were recorded at a range of applied potentials from 0 to +2000 mV (Figure 11, top). In the neutral state, the film appears brown, but becomes blue when doped. At potentials above +500 mV, absorption bands at *λ*=569 and 759 nm intensify, the latter as a result of the formation of polarons in the polymer chain. The sharp and prominent peak at *λ*=759 nm suggests that the polarons are localised within one region of the polymer chain, with a high probability that the bis-EDOT units are hosts for the cation radicals. At around +1500 mV, the bands at *λ*=337, 425, 569 and 759 nm diminish as a result of the generation of bipolarons and a rearrangement of the *π*-conjugated character in the polymer chain. The spectra recorded under the range of negative applied potentials are shown in Figure 11 (bottom). There is an increase in absorption in the wavelength range between *λ*=500 and 900 nm at voltages beyond −250 mV. This matches the peak seen in the polymer reduction (seen at around −1500 mV when referenced to ferrocene), and arises from the charge transfer of anion species between polymer chains. Due to the relatively high oxidation potential of poly(**15**), compared to PEDOT (polyethylenedioxythiophene) derivatives, together with the value of the band gap (1.7–1.8 eV) and the sharp nature of the spectroelectrochemical features, it is reasonable to assume that the conjugation path of the polymer is limited to nitrostyryl-thiophene-EDOT-EDOT-thiophene-nitrostyryl (acceptor–extended donor–acceptor) segments. This means that the **a** conformer has been frozen in the electropolymerisation experiment and that the conformer is persistent in the solid state.

**Computational studies**: All calculations were run by using Gaussian 03.^68^ Density functional theory calculations at the B3LYP/6-31G* level on the crystal structures of **13 a** and **13 b** showed that **13 a** is the lower-energy conformer, and the energy difference between the two forms was shown to be 70.2 kJ mol^−1^. The effect of bulk solvent was considered through the polarisable continuum model, by using a relative dielectric constant of *ε*_r_=4.9 for chloroform. This showed a small increased energetic preference for **13 a** with an energy difference of 73.4 kJ mol^−1^ between the two conformers.

**Figure 12:**
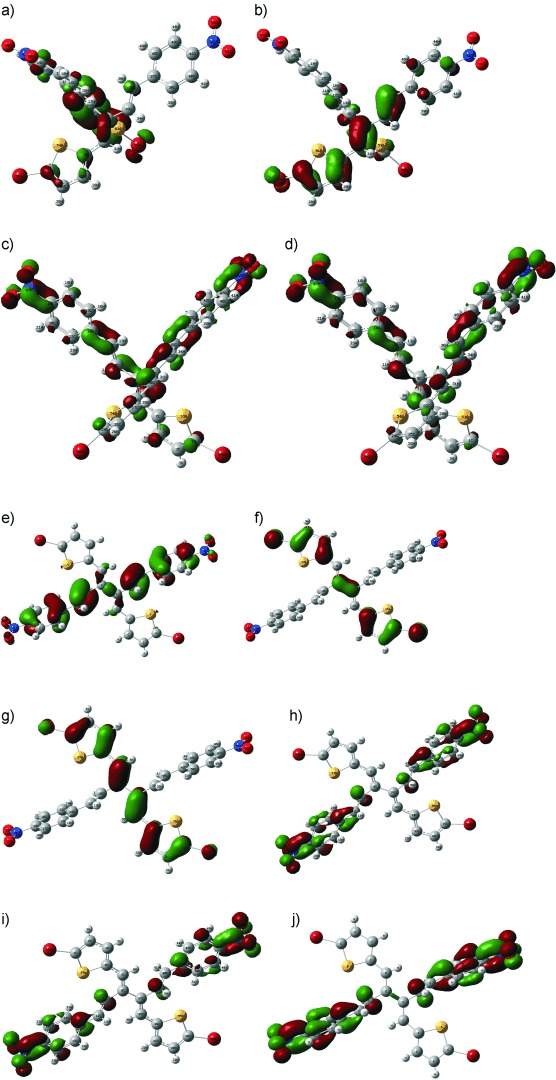
Selected molecular orbitals of 13 a (a–d) and 13 b (e–j) at isosurface values of 0.03, calculated by using Gaussian 03.^68^ a) HOMO−1, b) HOMO, c) LUMO, d) LUMO+1, e) HOMO−2, f) HOMO−1, g) HOMO, h) LUMO, i) LUMO+1 and j) LUMO+2.

Selected molecular orbitals of **13 a** and **13 b** at isosurface values of 0.03 are shown in Figure 12. In **13 a** the two highest energy occupied orbitals, HOMO−1 and HOMO are largely localised on one or the other of the donor–acceptor “arms”, with only a small contribution from the other arm. The LUMO and LUMO+1 levels of **13 a**, on the other hand, are evenly distributed between the two arms. Promotion of an electron from either HOMO−1 or HOMO to either of these unoccupied molecular orbitals, therefore, involves a component of transfer from one arm to the other. Electron transfer by this mechanism in **13 a** would, therefore, occur in the directions of the acceptor arms.

In contrast, the LUMO and LUMO+1 orbitals of **13 b** are localised along the same axis. Promotion to these orbitals will have the effect of transporting charge along this axis. The HOMO−2 and LUMO+2 orbitals of **13 b** represent *π*-bonding and antibonding components localised on the nitrostyryl arms. It is noteworthy that in HOMO−2 the calculations predict a degree of conjugation between the nitrostyryl units, through a central node. This is an unexpected finding given the twisted molecular geometry, marking this conformer as a potential model for a cross-conjugated mesomeric betaine.^69^

The eigenvalues (in eV) of the HOMO−1, HOMO, LUMO and LUMO+1 orbitals of **13 a** and **13 b** (calculated in the gas phase and with polarisable continuum model for chloroform) are shown in Table 1. In the gas phase and in the presence of chloroform there is a strong equivalence between the HOMO and HOMO−1 pair, and between the LUMO and LUMO+1 pair of **13 a**, as evidenced by the small energy difference between each pair of eigenvalues. The LUMO and LUMO+1 pair of **13 b** is also strongly equivalent. These small differences in eigenvalue probably originate from the use of the crystal structure data that do not precisely represent the optimised gas-phase geometry. It should be noted that there is only a small effect from incorporating the solvent dipole into the calculations.

**Table 1:** Eigenvalues of HOMO−1, HOMO, LUMO and LUMO+1 of 13 a and 13 b.

	13 a(gas phase)	13 a(CHCl_3_)	13 b(gas phase)	13 b(CHCl_3_)
HOMO−1 [eV]	−5.908	−5.665	−6.636	−6.458
HOMO [eV]	−5.842	−5.600	−5.557	−5.348
Δ*E* (HOMO−1/HOMO) [eV]	0.066	0.065	1.079	1.110
LUMO [eV]	−2.794	−2.703	−2.547	−2.493
LUMO+1 [eV]	−2.701	−2.650	−2.462	−2.425
Δ*E* (LUMO/LUMO+1) [eV]	0.093	0.053	0.085	0.069

The HOMO/HOMO−1 pair in **13 b** exhibit greater difference than in **13 a**. The HOMO (Figure 12 g) is localised on the bis-5-bromothiophene-1,4-butadiene segment, and follows the expected double-bond pattern for the ground state. The HOMO−1 (Figure 12 f) is also located over the same atoms but shows double-bond character between the two central carbon atoms. This difference is reflected in the eigenvalue difference of around 1.1 eV. For both forms, **13 a** and to a greater extent **13 b**, there is spatial separation between the HOMO/HOMO−1 and LUMO/LUMO+1 orbital pairs. Such an arrangement is desirable for tailoring the energy of either frontier orbital by molecular design.

Time dependant DFT calculations were performed to investigate the excited states of **13 a** and **13 b** in the gas phase and in chloroform. Table S1 and Table S2 show the first ten excited states of **13 a** and **13 b**, respectively. The excitation energy is shown, along with the dominant transition that is responsible for the excitation. That is, the electronic transition that has the highest wave function coefficient. The square of the wave function coefficient is the weighting of this transition to the overall excitation. The oscillator strength of the excitation is also shown, which relates directly to the strength of that band in the electronic absorption spectrum.

The excited state data can be used to interpret the UV/Vis spectrum of **13**. The first four excited states of **13 a** represent the longest wavelength absorptions in the spectrum. Transitions from the HOMO or HOMO−1 to the LUMO or LUMO+1 frontier orbitals shown in Figure 12 are the largest contributors to these four excitations, as evidenced by the coefficient of the wave function. The largest oscillator strength among these four excitations can be expected to dominate the appearance of the spectrum and give rise to the main bands. In the gas phase and the chloroform calculations for **13 a**, the largest value of the oscillator strength occurs for the fourth excited state transition, predominantly a HOMO−1→LUMO+1 transition. The energy of this excitation was calculated to be 411.91 nm in the gas phase, and 454.81 nm in chloroform. The experimental *λ*_max_ of the UV/Vis spectrum in chloroform was recorded as 404 nm.

(*E*,*E*)-1,4-Di(2′-thienyl)-1,3-butadiene **23** 
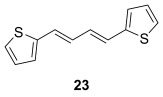
is analogous to the bis-bromothiophene butadiene *π* system in **13 b**. The spectroscopic properties of **23** have been reported elsewhere.^70^ The *λ*_max_ is reported at 381 nm in CH_2_Cl_2_. This wavelength is 14 nm shorter than the absorption maximum for **13 b**. At first inspection, the *π*–π* transition in **23** seems to be a good model for interpretation of the UV/Vis spectrum of **13 b**. A substituent effect, which is expected to be dominated by the strongly electron-withdrawing nitrostyryl units, could be invoked. A comparison between **22** and **13 a** shows that the difference in *λ*_max_ arising from the substituent effect of one donor–acceptor “arm” that is absent in **22** is 7 nm. Considering **23** and **13 b**, the dendralene absorption is affected by the presence of two nitrostyryl groups that are absent in **23**. Thus, the observed substituent effect is exactly double, which is shown by the comparison between **22** and **13 a**. However, the theoretical calculations on **13 b** predict that the largest value for the oscillator strength occurs for the 3rd excited state, which is predominantly a HOMO→LUMO+2 transition. The energy of this excitation was calculated to be 380.35 nm in the gas phase, and 396.16 nm in chloroform. This also coincides well with the experimental *λ*_max_ of the UV/Vis spectrum in chloroform (395 nm). The HOMO of **13 b** (Figure 12 g) is localised on the bisbromothiophene-butadiene unit, while the LUMO+2 orbital (Figure 12 j) is localised on the acceptor nitrobenzene units. It is surprising that this intramolecular charge transfer process is predicted in **13 b**, given the twisted geometry between these units. This process, which is predicted only to occur in solvated conditions, not the gas phase, must be a through-space event.

The only other significant oscillator strength value for transitions in **13 b** occurs for the transition HOMO−2→LUMO, predicted at *λ*=324 nm (CHCl_3_), or *λ*=306 nm (gas phase). This is in good agreement with the higher energy band seen at *λ*=320 nm in the UV/Vis spectrum of **13 b**. The Gaussian calculations predict that the HOMO−2 and the LUMO orbital of **13 b** are predominantly localised on the nitrostyryl arms. Therefore, there is reasonable agreement between theoretical and experimental results. The small observed shift to higher energy of the UV/Vis absorption maximum on conversion of **13 a** to **13 b** is reflected in the predicted largest absorption bands.

**Scheme 5:**
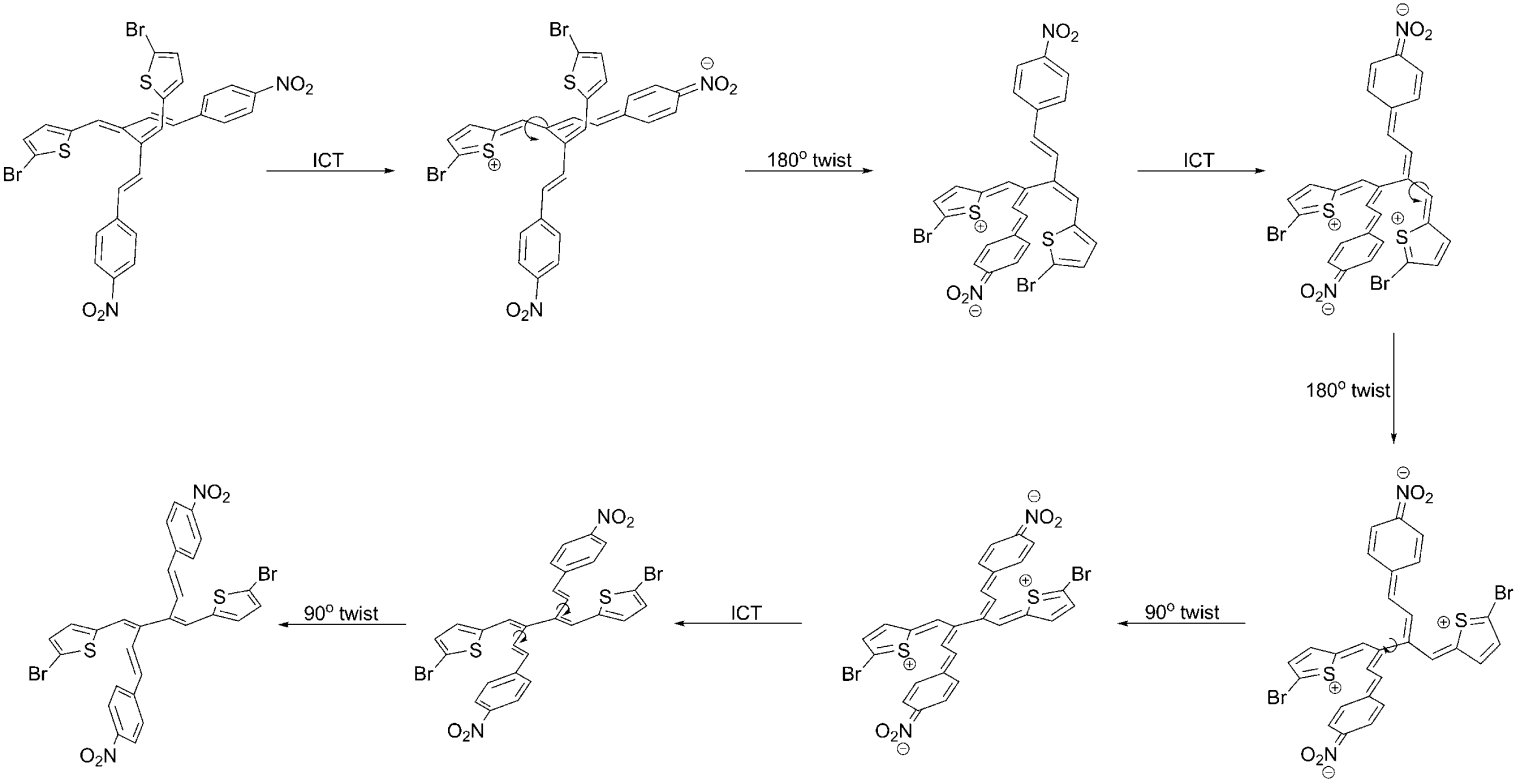
Proposed mechanism for the conversion of 13 a to 13 b.

**Mechanism for interconversion**: A mechanism for the conversion of **13 a** to **13 b** in solution is proposed in Scheme 5. The conformer **13 a** undergoes intramolecular charge transfer (ICT) by absorption of light to form a charge-separated excited state. The proposed quinoidal *π*-bonding in the excited state is supported by the computational modelling results, which show that promotion of electrons from the HOMO (Figure 12 b) and HOMO−1 (Figure 12 a) to the LUMO (Figure 12 c) or LUMO+1 (Figure 12 d) results in *π*-bond electron density localised on the appropriate C-C bonds. The newly formed single bonds confer additional rotational freedom on the molecule and the excited state structure is able to rearrange to another excited state geometry. Rotation of the bonds indicated with curved arrows gives rise to this structure in which the bond between the carbons α and β to the thiophenes are twisted to give a *syn* relationship between the donor–acceptor pair. The excited molecule is able to twist around the central C-C bond to form a planar structure. Relaxation to a neutral form is possible when the excited electrons return through the conjugated framework to the thiophene group, while the nitrobenzyl groups twist out of plane to reduce steric clash with the thiophene groups. This results in structure **13 b**.

Interestingly, structure **13 a**, which was the predominant conformer in the mixture that was first isolated (as identified by TLC and ^1^H NMR spectroscopy), is analogous to the structure of the precursor aldehyde **11**. It is reasonable to suggest that this donor–acceptor conformation is retained during the Wittig reaction, and kinetically stable under the reaction conditions (heating to reflux). In the solution state, ^1^H NMR indicates that the aldehyde **11** has the same conformation as the crystal structure. The NMR shows no change when the solution is exposed to ambient light over several days. This suggests that an aldehyde group is not a strong enough acceptor for the conformational change to occur.

## Conclusion

Two new [4]-dendralenes bearing thiophene–nitrostyryl donor–acceptor units have been prepared as mixtures of conformers. Pure samples of the conformers have been isolated by recrystallisation or flash column chromatography to allow the study of light-driven conformational transformations. In their application as electronic materials, the two types of conformers represent useful monomers for semiconductor conjugated polymer structures (**13 b** and **15 b**) or unusual conjugated donor–acceptor architectures for second order non-linear optics (**13 a** and **15 a**). The conversion of conformers **a** to **b** is facile and is initiated by photoexcitation, giving rise to a change in double-bond character between conjugated donor–acceptor pairs through an intramolecular charge transfer process. The reverse process is forbidden through the same mechanism since the donor–acceptor components are no longer conjugated in the **b** conformers. The single/double bonds remain localised in such molecules and this situation prohibits the key twist in the structure that is necessary for the regeneration of **a** conformers. Whilst through-space ICT does not allow switching from conformer **13 b** to **13 a**, the reverse step can be partially achieved by the application of heat. Controlling the conversion between two such very different conformers in polymeric structures would be extremely valuable in the field of organic electronics. From this viewpoint, these promising experiments will be expanded to answer the following questions: Can the reversibility of the **a**→**b** switch be improved by applying an alternative stimulus to heat (e.g. a redox process)? Are the kinetics of the **a**→**b** switch size-dependent? Can the transformation be applied to macromolecules? If these issues can be addressed favourably, then the realisation of conjugated poly(dendralene)-based switches will provide a new and fascinating direction for plastic electronics.

## Notes

Detailed facts of importance to specialist readers are published as ”Supporting Information”. Such documents are peer-reviewed, but not copy-edited or typeset. They are made available as submitted by the authors.
