# Supplementary material for: Controlling the Conformational Changes in Donor–Acceptor [4]-Dendralenes through Intramolecular Charge-Transfer Processes
Source: Chemistry. 2009 Sep 16;15(43):11581–93. doi: 10.1002/chem.200900656 (PMC3784042; doi:10.1002/chem.200900656)
Supplement: Supplementary file 1 [file chem0015-11581-SD1.pdf]

CHEMISTRY
A EUROPEAN JOURNAL

Supporting Information

© Copyright Wiley-VCH Verlag GmbH & Co. KGaA, 69451 Weinheim, 2009

Controlling the Conformational Changes in Donor-Acceptor [4]-Dendralenes Through Intramolecular Charge-Transfer Processes

A. L. Kanibolotsky, J. C. Forgie, G. J. McEntee, M. M. A. Talpur, P. J. Skabara, T. D. J. Westgate, J. J. W. McDouall, M. Auinger, S. J. Coles, M. B. Hursthouse

General.

Melting points were taken using a Stuart Scientific SMP1 Melting Point apparatus and are uncorrected. IR were recorded on Perkin Elmer Spectrum One FT-IR Spectrometer. ^{1}H and ^{13}C NMR spectra were recorded on a Bruker Avance / DPX400 at 400.13 and 100.61 MHz and Bruker Avance / DRX500 at 500 MHz and 125 MHz in $CDCl_3$, acetone-d_6 and DMSO-d_6; chemical shifts are given in ppm; all *J* values are in Hz. Elemental analyses were obtained on a Perkin Elmer 2400 analyzer. Electron absorption spectra were measured on a Unicam UV 300 spectrophotometer. Electrochemistry experiments were carried out with a CHI 660A (CH Instruments, Inc) and BAS-CV50W (Bioanalytical systems, Inc) electrochemical workstations with positive feedback compensation. Mass spectra were recorded on a Micromass VG platform II quadrupole mass spectrometer for atmospheric pressure chemical ionization (APCI) conditions. Accurate mass measurements were recorded on a Kratos Concept mass spectrometer.

X-Ray crystallography

X-ray diffraction data for compounds **13a, 13b, 11** and **22** were collected by means of combined phi and omega scans on a Bruker-Nonius KappaCCD area detector situated at the window of a rotating anode (λMo-k_{α} = 0.71073Å). The structures were solved by direct methods, SHELXS-97 and refined using SHELXL-97.[1] Hydrogen atoms were included in the refinement, but thermal parameters and geometry were constrained to ride on the atom to which they are bonded. The data were corrected for absorption effects using SADABS.[2] CCDC 723523 - 723526 contain the supplementary crystallographic data for this paper (for compounds **13a, 13b, 11** and **22**). These data can be obtained free of charge from The Cambridge Crystallographic Data Centre via www.ccdc.cam.ac.uk/data_request/cif

Electrochemistry

All experiments were carried out using ferrocene as the internal reference and potentials are quoted relative to the ferrocene/ferrocenium redox couple.

1,4-dibromodithiophene-2,3-diformylbutadiene (11)

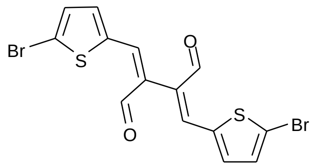

A mixture of 5-bromothiophene-2-carboxaldehyde (25.0 g, 0.131 mol), 2,5-dimethoxytetrahydrofuran (8.647 g, 0.0654 mol), Potassium acetate (6.421 g, 0.0654 mol), glacial acetic acid (10 ml) and water (10 ml) was refluxed for 16h then allowed to cool. The resultant dark brown mixture was added to water (100 ml), this mixture was extracted with ethyl acetate (3 x 100 ml). The combined organic extracts were washed with saturated aqueous sodium hydrogen carbonate (200 ml) and water (100 ml), heated gently for 15 min with a small amount of activated charcoal, and dried with magnesium sulphate. The solvent was removed in vacuo and the major product isolated by chromatography (silica, eluent 1:1 DCM:petroleum ether) as a light brown crystalline solid, 6.21 g, 22%. MP 180-185ºC; APCI –ve MS: 431; Accurate mass calculated for $C_{14}H_8O_2Br_2S_2$ 429.8327, found 429.8325; ^{1}H NMR (500 MHz, $CDCl_3$) δ_H (ppm): 9.68 (2H, s), 7.83 (2H, s), 7.21 (2H, d, J = 4.0 Hz), 7.07 (2H, d, J= 4.0 Hz); ^{13}C NMR (100 MHz, $CDCl_3$) δ_C / ppm: 191.01, 145.60, 138.76, 136.20, 136.16, 131.44, 131.37, 122.15; IR ν (cm^{-1}): 2822, 2360, 1678, 1620, 1588, 1508, 1428, 1413, 1248, 1233, 1151, 1108, 1061, 971, 909, 756; UV-vis, λ_{max} = 377 nm, ε = 24000; Anal. calculated for $C_{14}H_8O_2S_2Br_2$: C, 38.91; H, 1.87; Br, 36.98; found: C, 39.18; H, 1.58; Br, 36.88

5,5'-((1*E*,3*E*)-2,3-bis(4-nitrostyryl)buta-1,3-diene-1,4,diyl)bis(2-bromothiophene) (13)

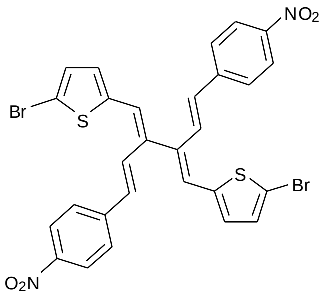

4-nitrobenzyltriphenylphosphonium bromide (8.855 g, 0.0185 mol) and potassium *t*-butoxide (2.076 g, 0.0185 mol) were mixed under nitrogen. THF (dry, distilled, 200 ml) was added by syringe to form a deep red suspension which was stirred for 2h at room temperature. A solution of 1,4-dibromodithiophene-2,3-diformylbutadiene **11** (2.0 g, 0.00463 mol) in THF (dry, distilled, 50 ml) was added by syringe and the mixture was refluxed under a nitrogen atmosphere for 48 hours. Silica gel was added to the crude reaction mixture and the THF was removed *in vacuo*. The residual mixture was added to a silica flash chromatography column and eluted with 3:1 petroleum ether:ethyl acetate. **13** was thus isolated as a mixture of conformers, 1.021 g crystalline red solid, 33%. MP: decomposed >250°; APCI –ve MS [M +2]: 670; Accurate Mass calculated for [M –Br]+ $C_{28}H_{18}O_4N_2BrS_2$ 588.9886 found 588.9883; Anal. calculated for $C_{28}H_{18}O_4N_2BrS_2$: C, 50.16; H, 2.71; N, 4.18; Br, 23.84; S, 9.57; found: C, 50.10; H, 2.47; N, 4.38; Br, 23.79; S, 9.17. Conformer **a** was isolated by recrystallization from dichloromethane-hexane, whilst conformer **b** was obtained by exposure of **13a** to ambient light.

Conformer 13a

^{1}H NMR (400 MHz, $CDCl_3$) δ_H (ppm): 8.16 (4H, d, J = 8.8Hz), 7.53 (4H, d, J = 8.8 Hz), 7.29 (2H, d, part obscured by solvent signal, J = 8.8 Hz); 7.23 (2H, s) 6.95 (4H, s) 6.46 (2H, d, J = 15.7 Hz); ^{13}C NMR (100 MHz, $CDCl_3$) δ_C (ppm): 146.9, 143.6, 140.3, 133.5, 132.8, 131.3, 130.9, 130.0, 128.1, 127.2, 124.3, 117.4; IR (cm^{-1}): 3093, 3003, 3906, 2824, 2648, 2439, 1578, 1508, 1420, 1335, 1182, 1108, 956, 864, 784, 734, 501; UV-vis, λ_{max} 404 nm, ε = 42000

Conformer 13b

^{1}H NMR (400 MHz, $CDCl_3$) δ_H (ppm): 8.21 (4H, d, J = 8.8 Hz), 7.71 (2H, dd, J = 15.7 Hz and 0.7 Hz), 7.57 (4H, d, J = 8.8 Hz), 7.11 (2H, d, J = 3.9 Hz), 6.94 (2H, d, J = 3.9 Hz and 0.7 Hz), 6.75 (2H, s), 6.71 (2H, d, J = 15.7 Hz), ^{13}C NMR (500 MHz, $CDCl_3$) δ_C (ppm): 147.8, 143.5, 141.0, 137.4, 132.7, 130.7, 130.6, 129.1, 127.5, 126.8, 124.4, 115.3; IR (cm^{-1}): 3076, 2926, 2657, 2442, 1730, 1582, 1505, 1423, 1316, 1183, 1109, 1059, 997, 861, 838, 790, 745, 689, 533; UV-vis, λ_{max} = 395, ε = 24000

5,5'-(5,5'-((1*E*, 3*E*)-2,3-bis(4-nitrostyryl)buta-1,3-diene-1,4-diyl)bis(thiophene-5,2-diyl))bis(3,4-ethylenedioxythiophene) (15)

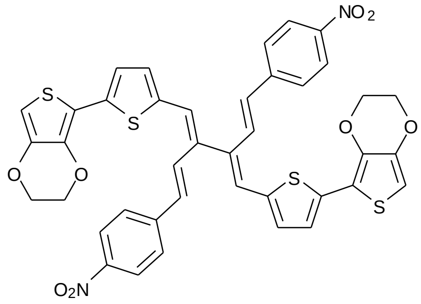

A mixture of 2-trimethylstannyl-3,4-ethylenedioxythiophene (as an approx. 60% mixture in 3,4-ethylenedioxythiophene, 0.097 g, 1.90 × 10^{-4} mol), **13a** (50 mg, 7.5 × 10^{-5} mol), $Pd(PPh_3)_4$ (0.018 g, 1.4 × 10^{-5} mol) and anhydrous DMF (2 ml) was heated in a microwave reactor for 60 min at 160 ºC. After cooling to room temperature the reaction mixture was added to brine (100 ml) and extracted with dichloromethane (100 ml plus 3 × 50 ml). The combined extracts were dried over sodium sulphate. The solvent was evaporated, and the crude mixture was subjected to column chromatography on silica gel eluting with a 7:3 mixture (v/v) of DCM:Petroleum ether, affording the product as a red solid, 0.043 g, 73%, MP 175-176 ºC; APCI –ve MS 793, Accurate mass calculated for $C_{40}H_{29}O_8N_2S_4$ 793.0801, found 793.0816; ^{1}H NMR (400 MHz, $CDCl_3$) δ_H (ppm): 8.12 (4H, d, J = 8.8 Hz); 7.50 (4H, d, J = 8.8 Hz); 7.34 (2H, d, J = 15.7 Hz); 7.30 (2H, s); 7.14 (2H, s); 7.13 (2H, s); 6.45 (2H, d, J = 15.7 Hz); 6.20 (2H, s); 4.28 (2H, t, J = 3.5 Hz); 4.21 (2H, t, J = 3.5 Hz); ^{13}C NMR (100 MHz, $CDCl_3$) δ_C (ppm): 146.5, 144.2, 142.0, 139.0, 138.3, 137.2, 134.5, 132.2, 131.5, 131.1, 127.0, 126.9, 124.1, 123.4, 112.1, 98.1, 77.4, 77.2, 76.9, 65.1, 64.6; IR (cm^{-1}): 3110, 2918, 2440, 2071, 1588, 1569, 1506, 1482, 1427, 1427, 1364, 1328, 1268, 1163, 1106, 1067, 1018, 966, 908, 859, 840, 790, 727; UV-vis, λ_{max} = 455 nm, ε = 89,000.

^{13}C NMR spectrum of compound **15**:

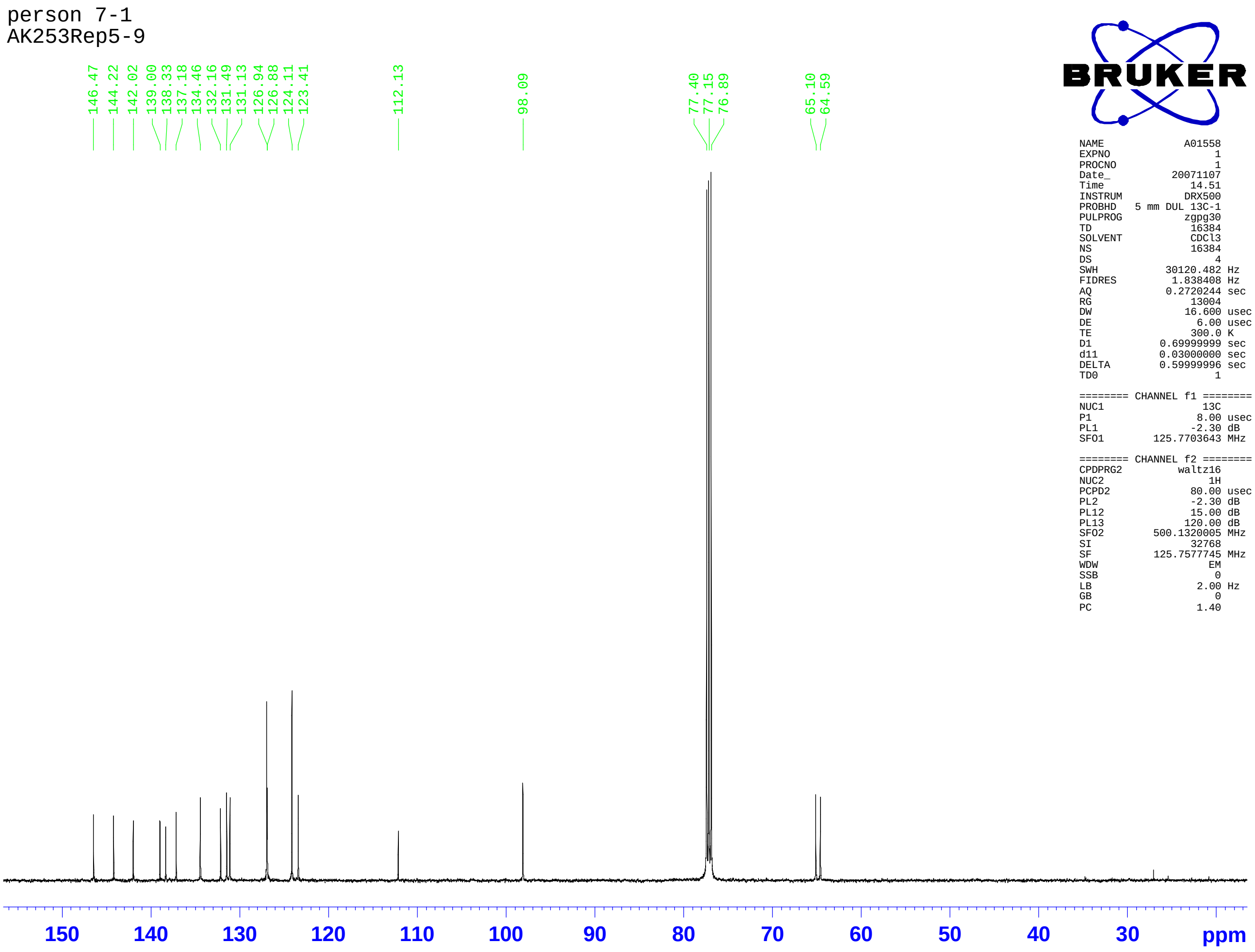

3-(thiophene-2-yl)acrolein (19)

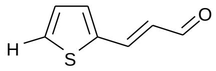

Lithium diisopropylamide (1.5M THF complex in cyclohexane, 16.6 ml, 0.0250 mol) was added dropwise over 10 min to a stirred solution of thiophene (1.98 ml, 4.07 g, 0.0250 mol) in freshly distilled dry THF (35 ml) under nitrogen at -78 ºC. After 45 min 3-(dimethylamino) acrolein (2.525 ml, 2.50 g, 0.0252 mol) was added and the mixture was allowed to room temperature then added to a 3 M aqueous solution of hydrochloric acid (200ml). This was extracted with dichloromethane (4 × 40 ml). The combined organic extracts were washed with a saturated aqueous solution of sodium hydrogen carbonate (50 ml) then water (50 ml), dried with magnesium sulphate and evaporated to yield a brown oil. This was dissolved in chloroform and filtered through a small plug of silica. The chloroform was removed in vacuo to yield a brown oil 0.635 g, 18%. APCI –ve MS 138; Accurate mass calculated for C_7H_7OS 139.0212, found 139.0211; ^1H NMR (500 MHz, $CDCl_3$) δ_H (ppm): 9.59 (1H, d, J = 7.7 Hz), 7.56 (1H, d, J = 15.6 Hz), 7.48 (1H, d, J = 5.0 Hz), 7.34 (1H, d, J = 3.5 Hz), 7.09 (1H, dd, J = 5.0 and 3.5 Hz), 6.47

(1H, dd, J = 15.6 and 7.7 Hz), ^{13}C NMR (125 MHz, $CDCl_3$) δ_C (ppm): 193.34, 144.92, 139.63, 132.62, 130.87, 129.03, 127.69; IR ν (cm^{-1}): 3105, 2819, 2723, 2361, 1672, 1613, 1420, 1360, 1227, 1119, 1046, 926, 858, 818, 712, 563.

3-(5-Bromothiophene-2-yl)acrolein (20)

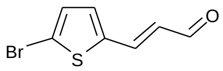

Lithium diisopropylamide (1.5M THF complex in cyclohexane, 16.6 ml, 0.0250 mol) was added dropwise over 10 min to a stirred solution of 2-bromothiophene (2.42 ml, 4.07 g, 0.0250 mol) in freshly distilled dry THF (35 ml) under nitrogen at -78 ºC. After 1 hour 3-(dimethylamino) acrolein (2.525 ml, 2.50 g, 0.0252 mol) was added and the mixture was allowed to room temperature then added to a 3 M aqueous solution of hydrochloric acid (200ml). This was extracted with dichloromethane (4 × 40 ml). The combined organic extracts were washed with a saturated aqueous solution of sodium hydrogen carbonate (50 ml) then water (50 ml), dried with magnesium sulphate and evaporated to yield a brown oil. Distillation using Kugelrohr apparatus (80 ºC, 0.15 mmHg) yielded a yellow brown oil 1.307 g, 6.02 mmol, 24%. APCI +ve MS: 217; Accurate mass calculated for C_7H_6OBrS 216.9317, found 216.9322; ^{1}H NMR ($CDCl_3$, 500 MHz) δ_H (ppm): 9.60 (1H, d, J = 7.6 Hz), 7.45 (1H, d, J = 15.7 Hz), 7.10 (1H, d, J = 3.9 Hz), 7.07 (1H, d, J = 3.9 Hz), 6.39 (1H, dd, J= 15.7 and 7.6 Hz), ^{13}C NMR ($CDCl_3$, 125 MHz): δ_C (ppm): 204.17, 143.59, 143.57, 132.80, 131.91, 127.81, 118.96.

1-(2-thieny1)-4(4-nitrobenzyl)buta-1,3-diene (21)

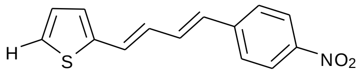

4-nitrobenzyltriphenylphosphonium bromide (0.320 g, 6.69 × 10^{-4} mol) was dissolved in dry, freshly distilled THF (15 ml) under nitrogen. Potassium tert-butoxide (0.075 g, 6.69 × 10^{-4} mol) was added, and the resulting orange/pink suspension was stirred under nitrogen for 30 min. A solution of aldehyde **62** (0.084 g, 6.08 × 10^{-4} mol) in dry, freshly distilled THF (10 ml) was added dropwise by syringe, and the mixture stirred under nitrogen for 16 hours. The resulting cherry-red solution with orange precipitate was added to water (50 ml), and this mixture was extracted with dichloromethane (3 × 50 ml). The combined organic extracts were dried over magnesium sulphate, filtered and evaporated to yield a bright orange microcrystalline solid. Column chromatography on silica with chloroform eluent afforded isolation of a fluorescent fraction which was identified by NMR as a mixture of cis- and trans- isomers. Recrystallization from dichloromethane and petroleum ether afforded the all-trans product as orange crystals.

0.030 g, 19 %. MP: 190-191 ºC; APCI –ve MS: 257; Accurate Mass calculated for $C_{14}H_{11}O_2NS$ 257.0505 found 257.0500; ^{1}H NMR (500 MHz, $CDCl_3$) δ_H (ppm): 8.19 (2H, d, J = 8.8 Hz), 7.53 (2H, d, J = 8.8 Hz), 7.26 (1H, d, J = 5.0 Hz), 7.08 (1H, d, J = 3.5 Hz), 7.03 (2H, m), 6.94 (1H, d, J = 15.5 Hz), 6.78 (1H, dd, J = 15.5 and 10.5 Hz), 6.67 (1H, d, J = 15.5 Hz); ^{13}C NMR (125 MHz, $CDCl_3$) δ_C (ppm): 146.95, 144.28, 142.62, 133.64, 130.23, 129.10, 128.36, 128.29, 127.60, 126.98, 126.03, 124.54; IR ν (cm^{-1}): 3108, 3010, 2916, 2829, 2691, 2438, 1583, 1505, 1426, 1341, 1245, 1182, 1139, 1108, 1079, 1042, 985, 962, 930, 883, 853, 829, 809, 745, 719, 689, 582, 532, 490; UV-vis, λ_{max} = 395, ε = 33000; Anal. calculated for $C_{14}H_{11}O_2NS$: C, 65.35; H, 4.31; N, 5.44; S, 12.46; found: C, 65.67; H, 4.09; N, 5.52; 12.77.

1-(2-bromo-5-thieny1)-4(4-nitrobenzyl)buta-1,3-diene (22)

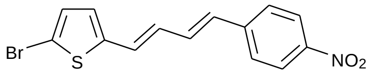

4-nitrobenzyltriphenylphosphonium bromide (3.168 g, 6.623 mmol) was suspended in dry, freshly distilled THF (140 ml) under nitrogen. Potassium tert-butoxide (0.743 g, 6.623 mmol) was added, and the resulting orange/pink suspension was stirred under nitrogen for 65 min. A solution of aldehyde **63** (1.307 g, 6.021 mmol) in dry, freshly distilled THF (30 ml) was added dropwise by syringe, and the mixture stirred under nitrogen for 21 hours. The resulting cherry-red solution with orange precipitate was added to water (250 ml), and this mixture was extracted with dichloromethane (3 × 100 ml). The combined organic extracts were dried over magnesium sulphate, filtered and evaporated to yield a bright orange microcrystalline solid. Column chromatography on silica with 3:1 petroleum ether:ethyl acetate eluent afforded isolation of a fluorescent fraction which was identified by NMR as a mixture of cis- and trans- isomers. Recrystallization from dichloromethane and petroleum ether afforded the all-trans product as orange crystals. 1.140 g, 56 %. MP 151-157 ºC; EI+ MS 337; Accurate mass calculated for $C_{14}H_{14}O_2N_2BrS$ 352.9954 found 352.9957; ^{1}H NMR (500 MHz, $CDCl_3$) δ_H (ppm): 8.19 (2H, d, J = 10.0 Hz), 7.53 (2H, d, J = 10.0 Hz), 7.00 (1H, dd, J = 15.4 and 10.4 Hz), 6.97 (1H, d, J = 3.8 Hz), 6.81 (1H, d, J = 3.8 Hz), 6.80 (1H, d, 15.4 Hz), 6.68 (1H, d, J = 15.4 Hz), 6.66 (1H, dd, J = 15.4 and 10.4 Hz), ^{13}C NMR (125 MHz, $CDCl_3$) δ_C (ppm): 147.32, 144.48, 144.31, 133.46, 131.44, 131.12, 128.96, 128.47, 127.76, 127.32, 124.82, 113.24; IR ν (cm^{-1}): 3088, 3034, 2915, 2825, 2439, 1591, 1509, 1426, 1381, 1352, 1324, 1238, 1115, 1056, 985, 959, 881, 855, 827, 787, 747, 691, 565, 534, 519, 486; UV-vis, λ_{max} = 397, ε = 43000; Anal. calculated for $C_{14}H_{14}O_2N_2BrS$: C, 50.01; H, 3.00; N, 4.17; Br, 23.77; S, 9.54; found: C, 49.94; H, 2.86; N, 4.21; Br, 23.96; S, 9.39.

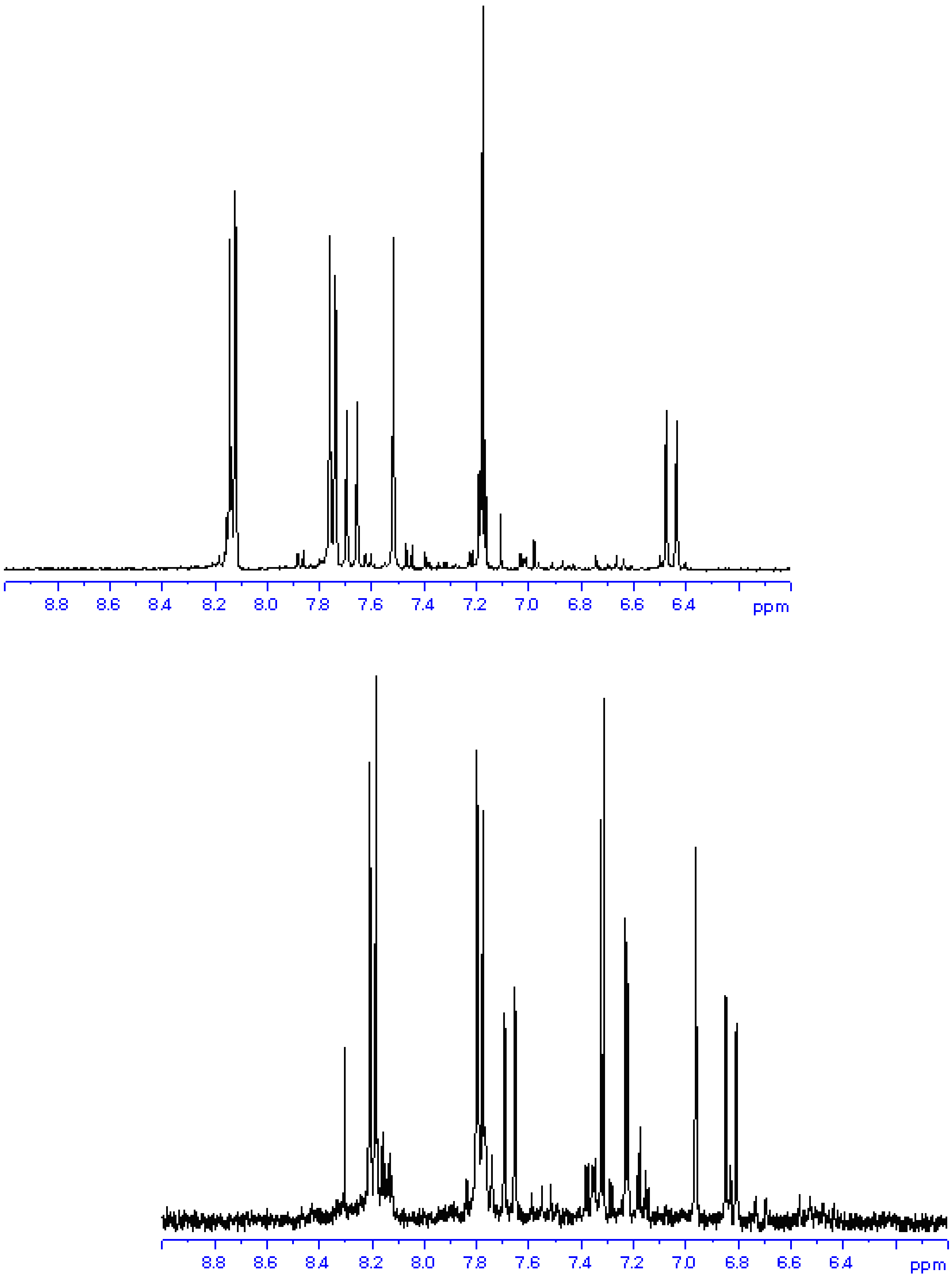

(a)

Figure S1. ^{1}H NMR spectra of (a) **13a** and (b) the solution after 24 h exposure to ambient light.

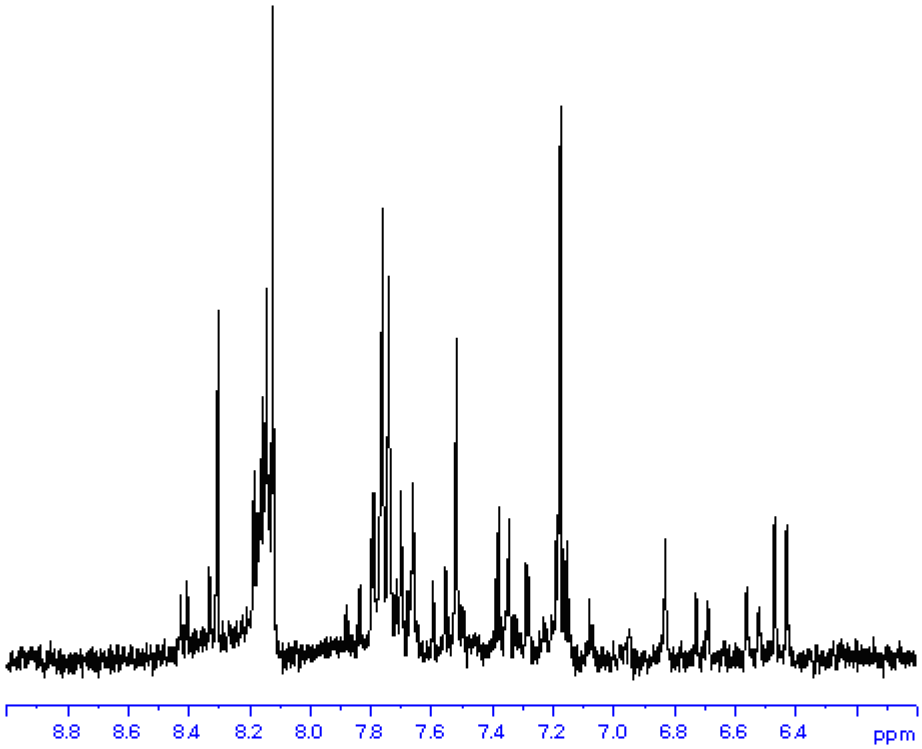

Figure S2. ^{1}H NMR of the solution from S1 (b) after microwave heating for 40 hours at 120 ºC.

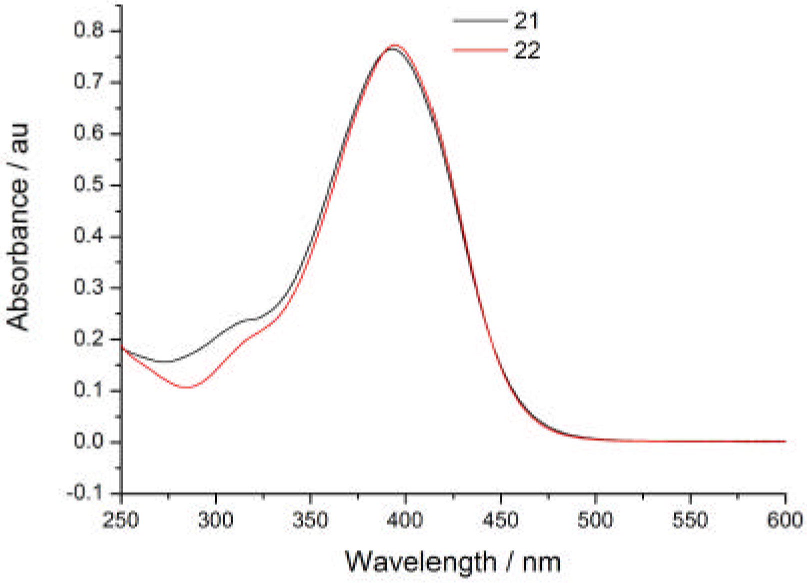

Figure S3. UV-visible absorption spectra of **21** and **22** in chloroform solution.

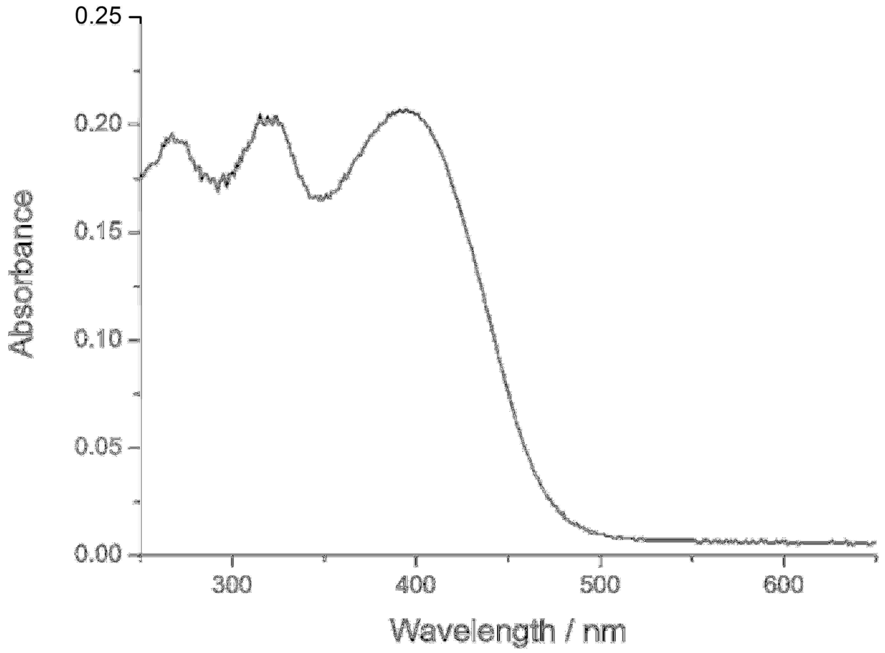

Figure S4. UV-visible spectum of **13b** in dichloromethane solution.

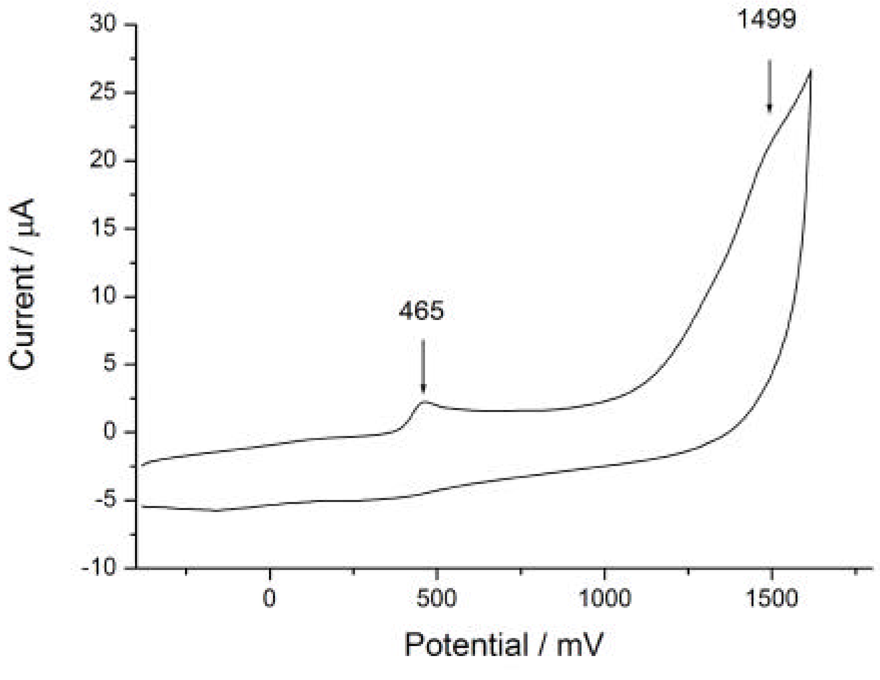

Figure S5. Cyclic voltammogram (oxidation) of **15** using a glassy carbon working electrode, Pt wire counter and silver wire pseudo electrodes, in CH_2Cl_2 (substrate *ca*. 10^{-4} M), *n*-Bu_4NPF_6 supporting electrolyte (0.1 M), scan rate 100 mV s^{-1}.

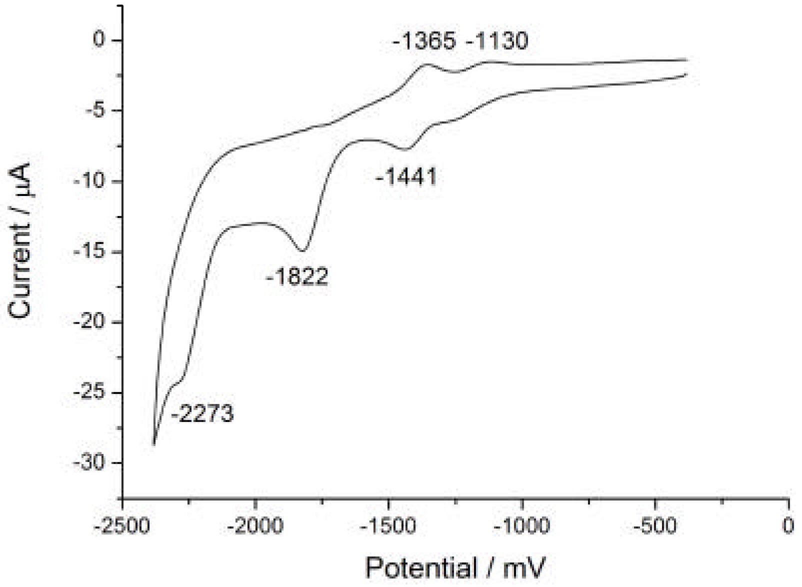

Figure S6. Cyclic voltammogram of **15** (reduction), using a glassy carbon working electrode, Pt wire counter and silver wire pseudo electrodes, in CH_2Cl_2 (substrate *ca*. 10^{-4} M), *n*-Bu_4NPF_6 supporting electrolyte (0.1 M), scan rate 100 mV s^{-1}.Note that the small wave at around -1300 mV is caused by reduction of oxygen in the solution..

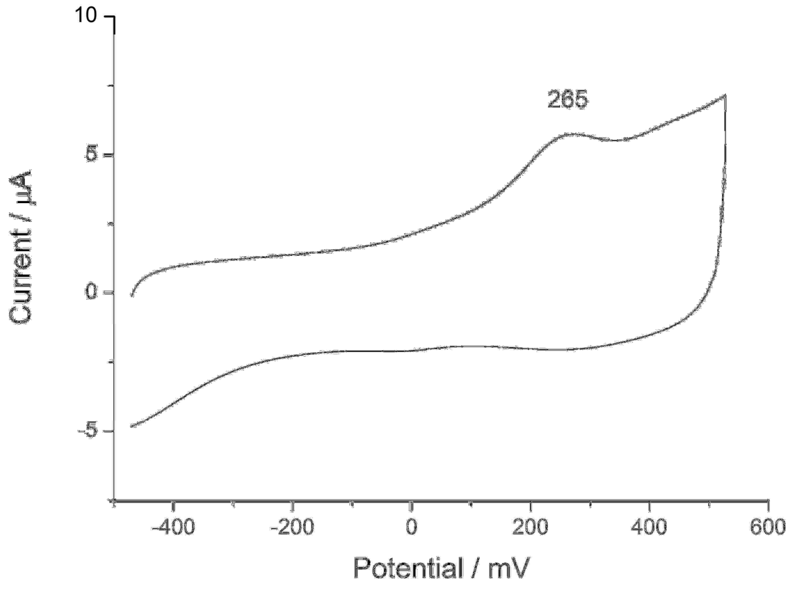

Figure S7. Oxidation of poly(**15**) deposited on a glassy carbon working electrode, using Pt counter and silver wire pseudo electrodes, in monomer-free acetonitrile containing *n*-Bu_4NPF_6 supporting electrolyte (0.1 M), scan rate 100 mV s^{-1}.

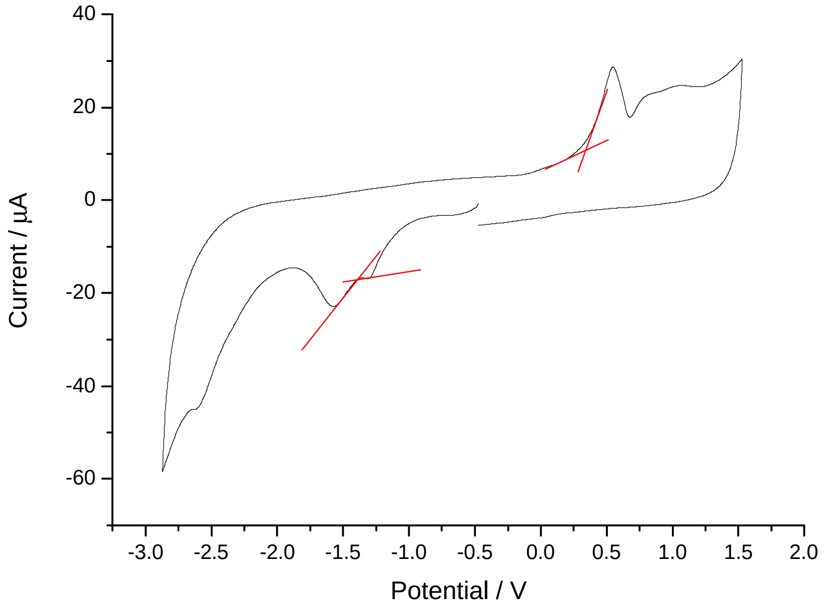

Figure S8. Reduction and oxidation of poly(**15**), showing onset of reduction and oxidation for determination of electrochemical band gap.

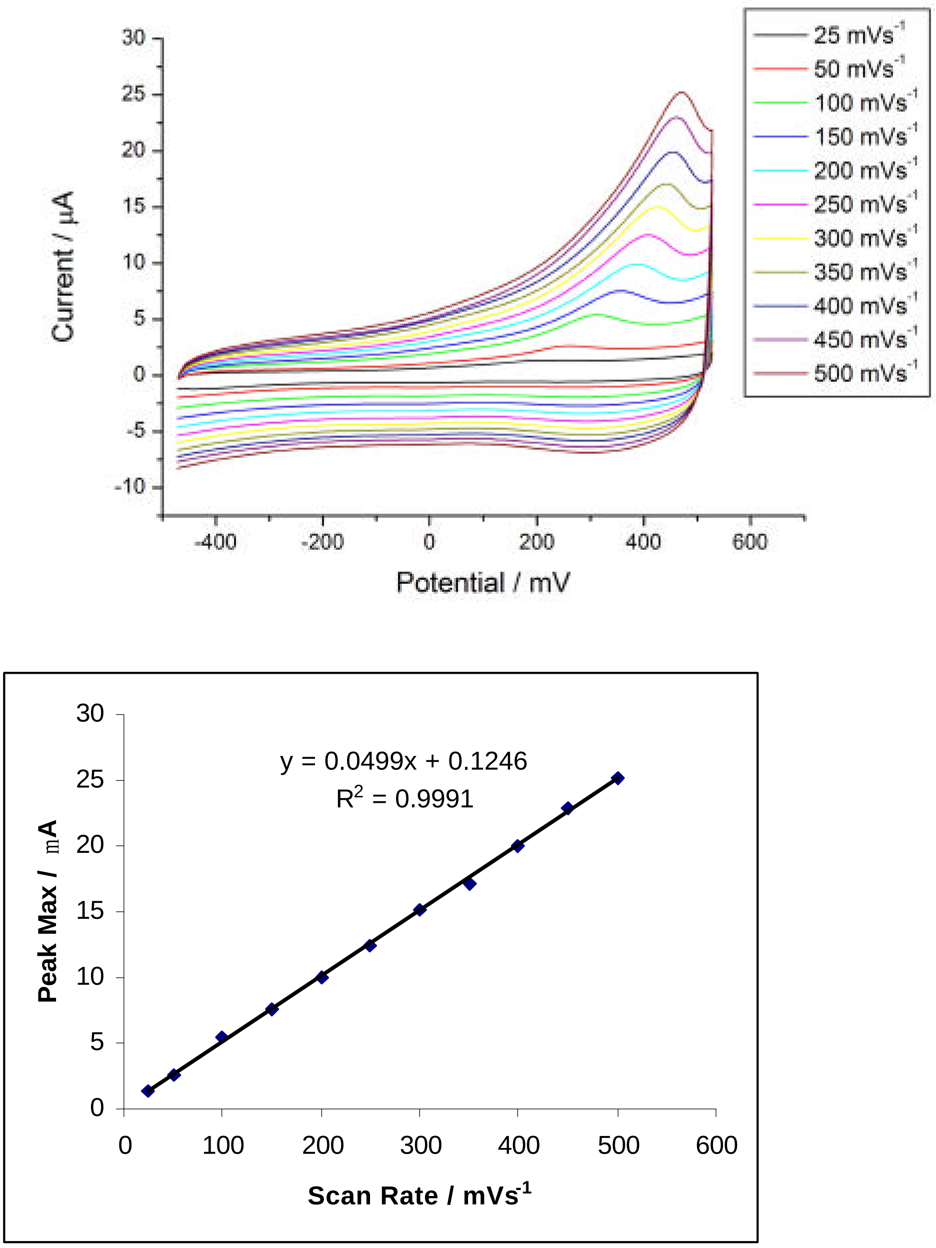

Figure S9. (Top) Oxidation of poly(**15**) at a range of scan rates, on glassy carbon working electrode, using silver wire pseudo reference and Pt counter electrodes, in monomer-free acetonitrile containing *n*-Bu_4NPF_6 supporting electrolyte (0.1 M); (bottom) plot of scan rate vs. maximum current of the oxidation peak.

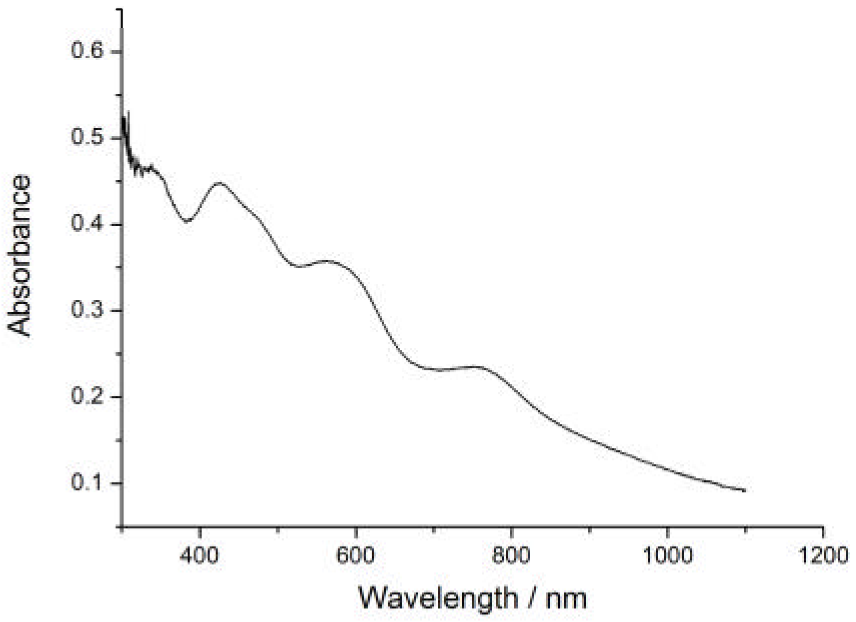

Figure S10. UV-visible spectrum of a doped film of poly(**15**) grown on ITO-coated glass.

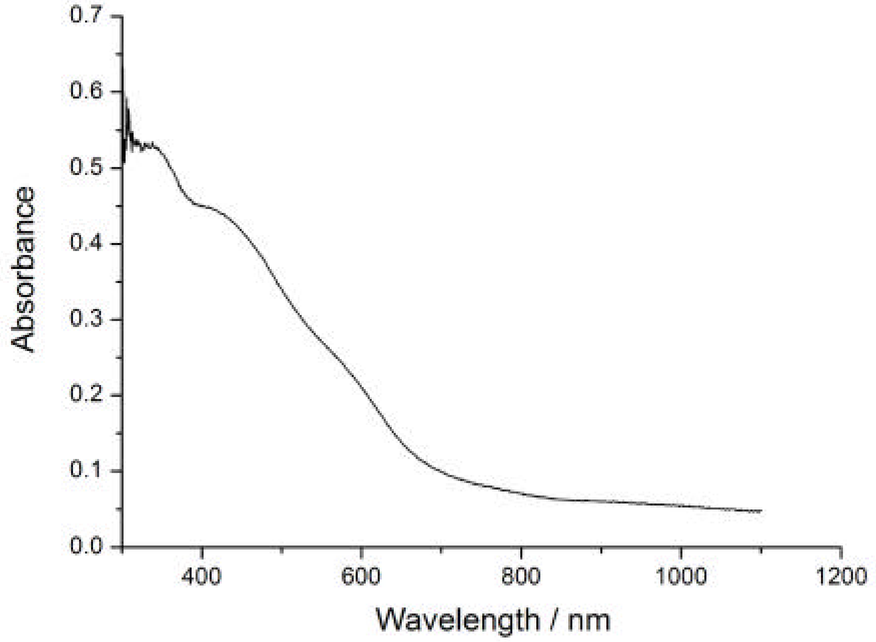

Figure S11. UV-visible spectrum of the de-doped film of poly(**15**), showing an estimation of the onset of the lowest energy peak for optical band gap determination.

Table S1. Excited states of **13a**.

**Excited State**	**Excitation energy (nm)**	**Dominant transition**	**Coefficient of wavefunction**	**Oscillator strength**
1 (gas phase)	478.47	HOMO → LUMO	0.517	0.0268
1 ($CHCl_3$)	502.28	HOMO → LUMO	0.549	0.0476
2 (gas phase)	470.84	HOMO-1 → LUMO	0.491	0.0211
2 ($CHCl_3$)	493.7	HOMO-1 → LUMO	0.475	0.0361
3 (gas phase)	419.23	HOMO → LUMO+1	0.502	1.0270
3 ($CHCl_3$)	462.54	HOMO → LUMO+1	0.509	1.0764
4 (gas phase)	411.91	HOMO-1 → LUMO+1	0.502	1.0670
4 ($CHCl_3$)	454.81	HOMO-1 → LUMO+1	0.549	1.0912
5 (gas phase)	353.22	HOMO → LUMO+2	0.567	0.0067
5 ($CHCl_3$)	371.67	HOMO → LUMO+2	0.574	0.0155
6 (gas phase)	348.31	HOMO-1 → LUMO+2	0.532	0.0043
6 ($CHCl_3$)	366.35	HOMO-1 → LUMO+2	0.549	0.012
7 (gas phase)	341.5	HOMO-8 → LUMO	0.378	0.0001
7 ($CHCl_3$)	341.43	HOMO → LUMO+3	0.548	0.5365

8 (gas phase)	339.38	HOMO-9 → LUMO+1	0.426	0.0009
8 ($CHCl_3$)	336.88	HOMO-1 → LUMO+3	0.525	0.5731
9 (gas phase)	328.03	HOMO → LUMO+3	0.518	0.3834
9 ($CHCl_3$)	327.33	HOMO-9 → LUMO+1	0.345	0.0085
10 (gas phase)	324.43	HOMO-1 → LUMO+3	0.481	0.4128
10 ($CHCl_3$)	326.91	HOMO-2 → LUMO	0.357	0.0080

Table S2. Excited states of **13b**.

Excited state	Excitation energy (nm)	Dominant transition	Coefficient of wavefunction	Oscillator strength
1 (gas phase)	486.54	HOMO → LUMO	0.699	0.0990
1 ($CHCl_3$)	511.86	HOMO → LUMO	0.701	0.0138
2 (gas phase)	468.54	HOMO → LUMO+1	0.701	0.0000
2 ($CHCl_3$)	495.75	HOMO → LUMO+1	0.703	0.0000
3 (gas phase)	380.35	HOMO → LUMO+2	0.62	1.3560
3 ($CHCl_3$)	396.16	HOMO → LUMO+2	0.637	1.5457
4 (gas phase)	338.43	HOMO-1 → LUMO	0.7	0.0000
4 ($CHCl_3$)	348.16	HOMO-1 → LUMO	0.701	0.0000
5 (gas phase)	329.69	HOMO-1 → LUMO+1	0.7	0.0142
5 ($CHCl_3$)	340.72	HOMO-1 → LUMO+1	0.702	0.0330
6 (gas phase)	322.66	HOMO-9 → LUMO	0.406	0.0006
6 ($CHCl_3$)	324.76	HOMO-2 → LUMO	0.67	0.9771
7 (gas phase)	322.62	HOMO-10 → LUMO	0.405	0.0005
7 ($CHCl_3$)	317.32	HOMO-2 → LUMO+2	0.609	0.0000
8 (gas phase)	312.11	HOMO-1 → LUMO+2	0.574	0.0000

8 ($CHCl_3$)	316.93	HOMO → LUMO+3	0.691	0.0132
9 (gas phase)	310.04	HOMO → LUMO+3	0.683	0.0332
9 ($CHCl_3$)	311.37	HOMO → LUMO+4	0.581	0.0000
10 (gas phase)	306.28	HOMO-2 → LUMO	0.64	0.7493
10 ($CHCl_3$)	310.54	HOMO-10 → LUMO	0.456	0.0000

Table S3. Data collection and refinement parameters for the crystal structures of **13a, 13b, 11 & 22**.

	**13a**	**13b**	**11**	**22**
Empirical formula	$C_{28}H_{18}Br_2N_2O_4S_2$	$C_{28}H_{18}Br_2N_2O_4S_2$	$C_{14}H_8Br_2O_2S_2$	$C_{14}H_{10}BrNO_2S$
Formula weight	670.38	670.38	432.14	336.20
Crystal system	Orthorhombic	Triclinic	Monoclinic	Monoclinic
Space group	*Pbca*	*P-1*	$P2_1/n$	$P2_1/n$
a (Å)	12.6788(2)	5.9961(2)	13.2503(3)	9.1236(3)
b (Å)	17.8990(4)	10.4376(5)	6.9083(2)	7.1185(3)
c (Å)	24.2841(4)	11.93116)	16.9813(5)	20.5445(7)
α (°)	90	109.211(2)	90.00	90.00
β (°)	90	91.493(3)	102.287(2)	94.055(2)
γ (°)	90	106.257(2)	90.00	90.00
Volume ($Å^3$)	5510.98(18)	671.16(5)	1518.81(7)	1330.95(8)
Z	8	1	4	4
Density (calc) (Mg/m^3)	1.616	1.659	1.890	1.678
Absorption coefficient (mm^{-1})	3.131	3.213	5.608	3.241
F(000)	2672	334	840	672
Crystal size (mm)	0.3 × 0.2 × 0.2	0.28 × 0.1 × 0.07	0.2 × 0.03 × 0.02	0.6 × 0.6 × 0.1
$θ_{max}$ (°)	27.5	27.48	27.5	27.49
Reflections collected	56764	11124	20077	14489
Independent reflections (I>2s I)	4560	2066	2828	2426
R(int)	0.0672	0.0363	0.0571	0.0457
Final R indices $F^2>2σF^2$	R1 = 0.0325<br>wR2 = 0.0572	R1 = 0.0395<br>wR2 = 0.0866	R1 = 0.0341<br>wR2 = 0.0648	R1 = 0.0373<br>wR2 = 0.0939
Δρ max / min ($eÅ^{-3}$)	0.345 / -0.508	0.248 / -0.497	0.375 / -0.622	0.510 / -0.878

References

[1] G. M. Sheldrick, 1997. SHELX97: Programs for structure solution and refinement, University of Göttingen, Germany.

[2] G. M. Sheldrick, 2003. SADABS. Version 2.10. Bruker AXS Inc., Madison, Wisconsin, USA.
